# Responses of the coral reef cryptobiome to environmental gradients in the Red Sea

**DOI:** 10.1371/journal.pone.0301837

**Published:** 2024-04-16

**Authors:** Rodrigo Villalobos, Eva Aylagas, Joanne I. Ellis, John K. Pearman, Holger Anlauf, Joao Curdia, Diego Lozano-Cortes, Alejandro Mejia, Florian Roth, Michael L. Berumen, Susana Carvalho

**Affiliations:** 1 King Abdullah University of Science and Technology (KAUST), Red Sea Research Center, Thuwal, Saudi Arabia; 2 The Red Sea Development Company, AlRaidah Digital City, Saudi Arabia; 3 School of Biological Sciences, Waikato University, Tauranga, New Zealand; 4 Coastal and Freshwater Group, Cawthron Institute, Nelson, New Zealand; 5 University of Seychelles and Blue Economy Research Institute Anse Royal, Victoria, Mahe, Seychelles; 6 Saudi Aramco, Environmental Protection Department, Dhahran, Saudi Arabia; 7 Stockholm University, Baltic Sea Centre, Stockholm, Sweden; King Abdulaziz University, SAUDI ARABIA

## Abstract

An essential component of the coral reef animal diversity is the species hidden in crevices within the reef matrix, referred to as the cryptobiome. These organisms play an important role in nutrient cycling and provide an abundant food source for higher trophic levels, yet they have been largely overlooked. Here, we analyzed the distribution patterns of the mobile cryptobiome (>2000 μm) along the latitudinal gradient of the Saudi Arabian coast of the Red Sea. Analysis was conducted based on 54 Autonomous Reef Monitoring Structures. We retrieved a total of 5273 organisms, from which 2583 DNA sequences from the mitochondrially encoded cytochrome c oxidase I were generated through sanger sequencing. We found that the cryptobiome community is variable over short geographical distances within the basin. Regression tree models identified sea surface temperature (SST), percentage cover of hard coral and turf algae as determinant for the number of operational taxonomic units present per Autonomous Reef Monitoring Structures (ARMS). Our results also show that the community structure of the cryptobiome is associated with the energy available (measured as photosynthetic active radiation), sea surface temperature, and nearby reef habitat characteristics (namely hard corals, turf and macroalgae). Given that temperature and reef benthic characteristics affect the cryptobiome, current scenarios of intensive climate change are likely to modify this fundamental biological component of coral reef functioning. However, the trajectory of change is unknow and can be site specific, as for example, diversity is expected to increase above SST of 28.5°C, and with decreasing hard coral and turf cover. This study provides a baseline of the cryptobenthic community prior to major coastal developments in the Red Sea to be used for future biodiversity studies and monitoring projects. It can also contribute to better understand patterns of reef biodiversity in a period where Marine Protected Areas are being discussed in the region.

## Introduction

Coral reefs shelter close to one-third of the ocean’s biodiversity, yet they represent less than 0.2% of the surface of the ocean’s floor [1–3]. Most of the biodiversity of organisms in coral reefs results from the cryptobiome [4], which is composed of species of small-size organisms inhabiting crevices within the reef matrix [2, 4, 5]. Their biomass per square meter can reach values one order of magnitude higher than that reported for zooplankton [6, 7], hence providing not only a diverse but an abundant food source for higher trophic levels [5, 8]. Cryptic assemblages incorporate diverse trophic categories, such as primary consumers [9, 10], detritivores, and predators [11, 12], which all interact in nutrient cycling in the reef system [6]. However, despite their diversity and importance in the functioning of benthic habitats, limited knowledge exists about the distribution and abundance patterns of the cryptobiome.

Within the cryptobiome, different species have specific niche preferences resulting from their differential sensitiveness to a range of environmental variables and biological interactions, which determine their distribution patterns [13, 14]. Among the factors that have been pointed out as driving biogeographic patterns, here we will focus in three of them: sea surface temperature, primary production and the characteristics of the reef habitat. Sea surface temperature (SST) is suggested as one of the most relevant [15–20]. The critical role of SST is increasingly reported for different marine species in light of global climate change [17, 21–24]. We have also been assisting at the increase of SST and the duration of heat stress events, with coral reefs suffering more frequent and more intense bleaching [25]. Temperature affects the performance, energy assimilation, and reproductive capacity of organisms [26–30]. Also, it has been hypothesized that energy transfers faster between trophic groups with increasing temperatures [31, 32].

Coral reef primary production is based on phytoplankton, benthic algae, and zooxanthellae in symbiosis with corals [33]. The abundance and biomass of primary producers are influenced by the position in the shelf, the oceanographic characteristics experienced at a reef [34], the energy available as photosynthetic active radiation (PAR), and nutrient availability affecting higher trophic levels through a bottom up effect [31, 32, 35, 36]. However, exceptions occur in pristine atolls with steep walls, which present a top-down control from an inverted pyramidal trophic structure [34]. Chlorophyll-a concentration is commonly used as a proxy of pelagic primary production [37]. Coral reefs as other marine ecosystems rely on phytoplankton for energy input [38]. For example, hard corals depend partially in heterotrophic feeding from plankton [39]. Some corals such as *Montipora capitata* are able to sustain all their physiological needs from heterotrophic feeding when needed [40]. Also, assessing PAR has been recommended to be used as a proxy to estimate the energy available for primary production [35], particularly between reefs with similar geomorphology and oceanographic characteristics.

A third factor that is critical for the distribution patterns of the cryptobiome is the availability of suitable substrates for settlement and colonization. Studies have reported that cryptobenthic fish assemblages are more abundant and speciose in coral rubble within the coral reef [41], accounting for almost half of the reef fish diversity [42]. Gastropods of the family Epitoniidae utilize fungid corals as a source of food and shelter, and can be found almost exclusively underneath their host [43]. The flatworm *Amakusaplana acroporae* is a corallivore found in *Acropora* colonies. There are also records of host-specificity, such as the case of crustaceans of the families Trapeziidae, Tetraliidae, Pontoniinae, and Alpheidae, known as symbionts or parasites of some corals or sponges [44–46]. In the Red Sea, several species of copepods of the genus *Spaniomolgus* are also known to inhabit shallow-water stony corals [47]. Species using habitats in proximity to artificial reef structures are likely to colonize the vacant surfaces [48], and even though the composition and structure of artificial and natural reefs may differ [49], the characteristics of the nearby habitat will most likely play a critical role on establishing assemblages [50].

The preferential residence in hidden spaces of the cryptobiome, their small size, and the lack of standardized approaches has limited the investigation of this diverse group of organisms in the past. In the last decade though, Autonomous Reef Monitoring Structures (ARMS) developed during the Census of Marine Life (CoML), contributed to an increasing number of studies performed under standardized conditions that will boost the knowledge of the biodiversity and ecological patterns of the cryptobiome [51–58]. ARMS units consist of stacked Polyvinyl Chloride (PVC) layers with spaces in between, either allowing or limiting water flow. The space between the PVC plates provides substrate and shelter to mobile and sessile cryptic organisms. Also, plates are differentially exposed to light. Overall, ARMS mimic the structural complexity of the reef and the diversity of ecological niches available for colonization, while providing a standardized way to quantify the cryptobiome. According to the standard protocol for the use of ARMS [53], the organisms are separated into three mobile fractions based on their size (100–500 μm, 500–2000 μm, >2000 μm) and one sessile fraction. Each fraction is composed of a different arrangement of organisms [4]. Overall, fractions are analyzed based on metabarcoding techniques (i.e., the whole fraction is blended and then analyzed through amplicon sequencing). The only exception is the largest mobile fraction (>2000 μm) that is analyzed based on a combination of morphological and molecular (i.e., Deoxyribonucleic acid (DNA) barcoding) techniques. This strategy allows quantifying the abundance, which is currently not achievable using metabarcoding technique, yet essential for assessments of ecosystem health [59, 60]. Therefore, to understand better how some of the characteristic organisms of the reef cryptobiome respond to changes in environmental patterns, this study is focused on the largest fraction of the mobile cryptobiome (i.e., >2000 μm).

Here we investigate the responses of coral reef cryptic organisms to key environmental variables that are known to potentially influence their distribution patterns. Given the high levels of diversity of this biological component of reef systems, different organisms are likely to have varying tolerances for abiotic variables [54]. The strong environmental gradients observed across the latitudinal extent of the Red Sea provide an excellent natural laboratory to examine the responses of the cryptobiome to some fundamental environmental variables (e.g., SST and chlorophyll-a) [61]. Changes along the latitudinal gradient allow identifying regions of contrasting environmental scenarios to test their combined effect in the distribution patterns of cryptic reef communities. The environmental gradient and standardized methodology of the ARMS in sampling the cryptobiome enabled several hypotheses to be tested: 1) Species richness of the cryptobiome will have a positive relationship to mean annual temperatures, as increased temperatures facilitate the transfer of energy between trophic levels in ectotherms; 2) Community composition will change along the temperature gradient; 3) Higher levels of primary production (using Chla and PAR as proxies) will result in higher levels of species richness and abundance in the cryptobiome; 4) The cryptobiome will be influenced by the characteristics of nearby reef habitats, as each habitat provide a specific array of niches with associated cryptic assemblages.

## Materials and methods

### Study area and sampling design

**Fig 1:**
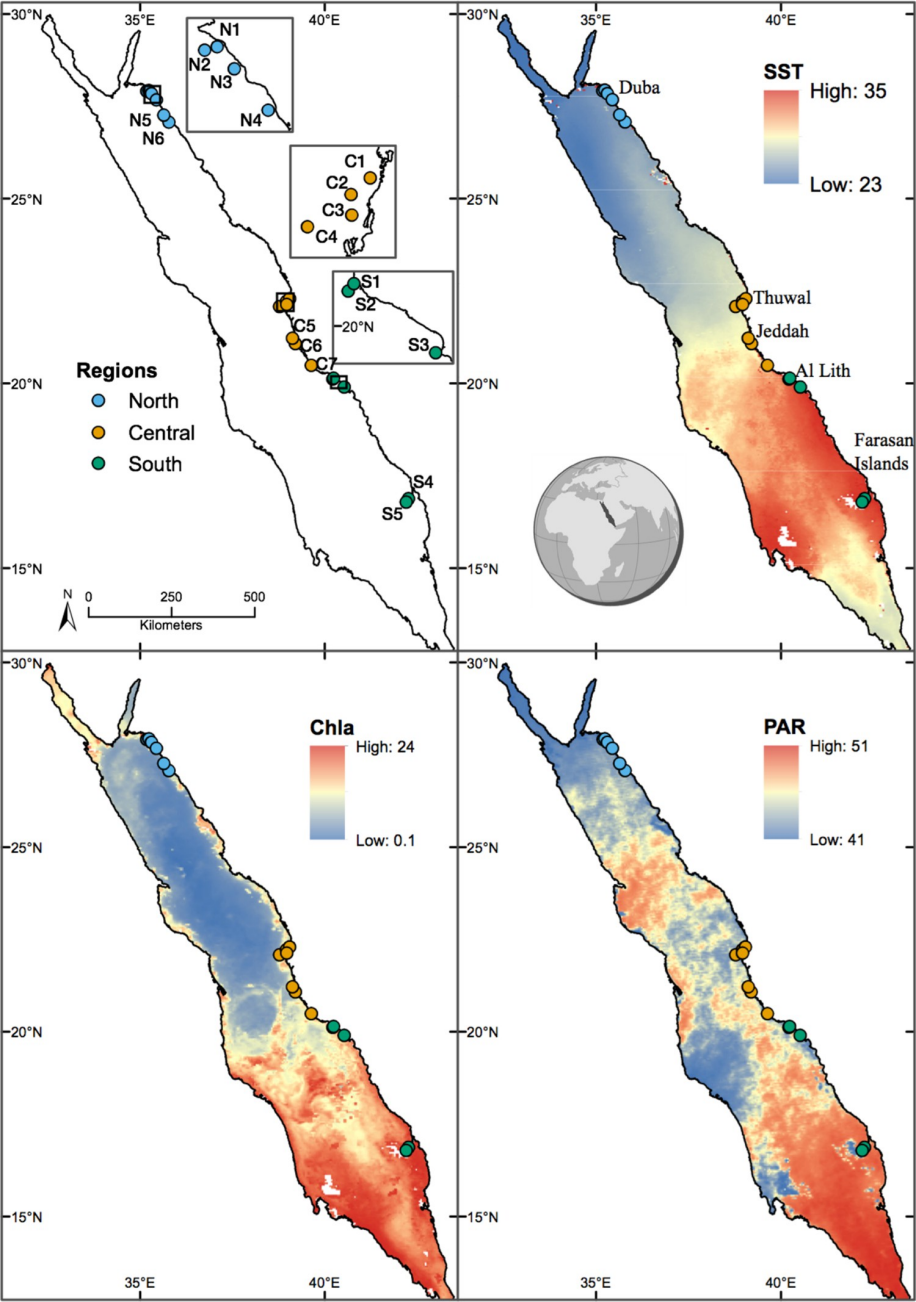
Reef sites sampled along the East Red Sea coast (Saudi Arabia). Top left figure showing the sites and their respective region (North, Center, South). Top right figure shows the average of sea surface temperature in Celsius (SST) from May 2015 to May 2017, i.e., during most of the deployment periods. The bottom left, and bottom right figure shows the average for the same time interval for Chlorophyll-a in mg m^-3^ (Chla) and photosynthetic active radiation (PAR) in Einstein m^-2^ d^-1^, respectively. Maps were designed using ArcMap (Version 10.7.1.), Environmental Systems Research Institute, Inc., Redlands (esri.com) by Ute Langner. SST data was obtained from NASA Goddard Space Flight Center, Ocean Ecology Laboratory, Ocean Biology Processing Group.

The Red Sea coral reef system extends along its main length from 12° 40’ 30”N to 28° 0’ 0”N [62]. The Red Sea presents fringing reefs along its coast [63]. In the central Red Sea patchy and barrier reefs are also present, increasing the type of coral communities [63]. The south Red Sea benthic community differs from the central and north Red Sea, with the major contribution to dissimilarity given by soft corals, rubble, sand, and coralline algae [64]. The reefs experience a gradual increase in SST from north to south, while the opposite trend is valid for salinity [65]. Chlorophyll-a (Chla) concentrations do not follow a consistent latitudinal gradient being affected by seasonal oceanographic patterns [65]. In general, the southern reefs experience higher levels of Chla driven by the intrusion of nutrient rich Gulf of Aden Intermediate Water (GAIW) especially in the spring and summer [66]. In the northern Red Sea, seasonal mixing of the water column in the winter can bring nutrients into the photic zone and an increase in primary productivity [67]. Along the Saudi Arabian Red Sea coast, 18 reefs were selected, across 11 degrees of latitude (Fig 1, S1 File). Reefs were divided into three regions according to the regional distinction made by [68], based on differences in temperature, nutrients, productivity, and connectivity. Six reefs, located in the vicinity of Duba (27° N; N1-N6) were assigned to the northern region. This region is characterized by lower SST and PAR than the central and southern regions. Seasonal variability in Chla concentrations is observed as vertical mixing of the water column in winter, brings nutrients into the photic zone and subsequently increases primary productivity [69]. Seven reefs were located in the central Red Sea close to Thuwal and Jeddah (22° and 20° N; C1-C7). This region is characterized by intermediate values of SST and PAR compared to the other regions. In addition, the central Red Sea maintains an oligotrophic profile throughout the year [70]. Five reefs were located in the southern Red Sea close to Al Lith and at the Farasan Islands (20° and 16° N; S1-S5), associated with the highest SST, PAR, and Chla concentrations [69].

Three ARMS replicates (54 in total) were placed in each selected reef at approximately 10 m depth on an hard substrate area; replicate units were separated by two to five meters. Units were deployed between June 2014 and May 2015 and recovered approximately two years later. Due to logistical constraints, 12 units (reefs JD02, JD03, R018, R024) could only be recovered three years after deployment. Coordinates, characterization of the reefs, deployment and recovery periods are provided in the S1 File.

Data on the benthic structure was generated from standard photo-transect surveys conducted at the time and depth of deployments. The surveys included triplicate transects of 20 m length (separated by 5m) and 1 m wide, with photos (1 x 1 m) taken every 2 m using the method described by [54]. Benthic groups were then identified using Coral Point Count with Excel extensions for 48 randomly distributed points [71]. From the benthic survey dataset, we used the following benthic categories: percentage cover of hard corals (HC), soft corals (SC), turf algae (Turf), macroalgae (MA), and not living substrates (named Abiotic for practicality in the following text; i.e., sum of sand, rubble, dead coral colonies, and rock).

Moderate-resolution Imaging Spectroradiometer (MODIS) Aqua Level-3 Mapped 11μm Day/Night Sea Surface Temperature, Version 2014 Data; NASA OB.DAAC, Greenbelt, MD, USA. doi: 10.5067/AQUA/MODIS/L3M/SST/2014; Accessed on 02/19/2020. Chla data was obtained from NASA Goddard Space Flight Center, Ocean Ecology Laboratory, Ocean Biology Processing Group. Moderate-resolution Imaging Spectroradiometer (MODIS) Aqua Level-3 Mapped Chlorophyll, Version 2018 Data; NASA OB.DAAC, Greenbelt, MD, USA. doi: 10.5067/AQUA/MODIS/L3M/CHL/2018; Accessed on 02/19/2020. PAR data was obtained from NASA Goddard Space Flight Center, Ocean Ecology Laboratory, Ocean Biology Processing Group. Moderate-resolution Imaging Spectroradiometer (MODIS) Aqua Level-3 Mapped Photosynthetically Available Radiation, Version 2018 Data; NASA OB.DAAC, Greenbelt, MD, USA. doi: 10.5067/AQUA/MODIS/L3M/PAR/2018; Accessed on 02/19/2020.

### Autonomous Reef Monitoring Structure–retrieval and processing

The ARMS units consist of nine PVC square plates (0.225 m x 0.225 m x 0.066 m) stacked on top of each other with spaces of 0.0128 m in between. Water flow is blocked in alternate spaces with bars forming a cross shape. The array of PVC plates is fixed to a base of 0.45 m by 0.35 m with weights to stabilize the ARMS at the sea floor. The ARMS target small invertebrates which commonly inhabit the reef matrix. Animals were sedated after collection with diluted magnesium chloride or diluted clove oil.

Before collection, each ARMS was covered in situ with a 106 μm mesh windowed plastic bin to avoid the loss of mobile specimens, while allowing water flow. Once aboard the boat, each ARMS was placed in its individual container with filtered local seawater (106 μm). In the lab, each ARMS was disassembled inside its container, and the plates were gently brushed on each side to detach mobile organisms to the filtered seawater. A visual inspection was done to each plate to pick by hand each mobile specimen left attached to the plate and placed back in the container. The water was then sieved with a 2000 μm sieve to collect larger mobile organisms. The material retained in the sieve was sorted and identified to the lowest taxon possible. A photographic record was kept for each organism. Each organism was assigned a label and stored in 80% ethanol for future DNA barcoding.

### Ethics statement

This study was undertaken according to guidelines established for sampling at King Abdullah University of Science and Technology (KAUST). No special authorization was required as the research did not include protected or endangered species. Research permits for sampling in Saudi Arabian waters were obtained from the Saudi Arabian coastguard. At the time of sampling, no guidelines were in place to regulate work not targeting vertebrates. Therefore, we could not obtain ethics approval or waiver.

### DNA extraction and DNA barcoding

A tissue sample (10 to 50 μg) was obtained from 5273 vertebrate and invertebrate organisms. Where possible, the taxonomically relevant traits were preserved during tissue sample collection. The DNA was then extracted using the DNeasy kit (Qiagen) as per the manufacturer’s instructions. Between 2 and 10 ng of each DNA extract was used for DNA barcoding. A 658 bp region of the mitochondrially encoded cytochrome c oxidase I (MT-CO1) gene was amplified using the primer combination jgLCO1490 (TITCIACIAAYCAYAARGAYATTGG) and jgHCO2198 (TAIACYTCIGGRTGICCRAARAAYCA) [72]. Polymerase chain reaction (PCR) was performed in a total reaction volume of 19 μL that consisted of 10 μL of GoTaq G2 Hot StartMaster Mix (Promega), 0.6 μL of each primer at 10 μM, 0.2 μL of 20 mg mL^-1^ bovine serum albumin (BSA), and 1 μL of extracted DNA. The thermocycling profile consisted of an initial denaturation step at 95°C for 5 min followed by 4 cycles of 94°C-30 s, 50°C-45 s, and 72°C-1 min, and by 34 cycles of 94°C-30 s, 45°C-45 s and 72°C-1 min, and a final 8 min elongation phase at 72°C [53]. PCR products were examined on 1.5% agarose gels stained with 4 μL of SYBR^TM^ Safe DNA gel stain per 100 mL. When amplification with the jgLCO1490—jgHCO2198 primer combination failed, the PCR was repeated using the LoboF1 (KBTCHACAAAYCAYAARGAYATHGG) and the LoboR1 (TGRTTYTTYGGWCAYCCWGARGTTTA) [73] primer combination keeping the PCRmix and conditions described. PCR products were purified using 2 μL of Illustra^TM^ ExoProStar^TM^ 1-step from GE Healthcare for 8 μL of PCR product. The PCR product was sequenced in Sanger ABI 3730 capillary platform using 5 μL of primer at 20 pmol and 10 μL of purified PCR product at the King Abdullah University of Science and Technology, Bioscience Core Laboratory (BCL).

From the 5273 organisms collected, a total of 2583 successful sequences were obtained. Forward and reverse reads obtained from the capillary platform were assembled using Geneious (Biomatters) with edges trimmed where the chance of a base error was greater than 5%. Sequences were discarded if a stop codon was present in the translated sequence to the invertebrate mitochondrial code or had three ambiguous bases that caused a wrong amino acid translation. Chordates sequences were filtered using the vertebrate mitochondrial code. All sequences were aligned and primers trimmed from each sequence. The phylogenetic tree was investigated for anomalously long branch lengths when compared to neighbouring taxa, which could indicate contamination. For example, a sequence of a galatheid which showed a branch twice as long compared to all other galatheid sequences would have been considered anomalously long. Operational taxonomic units (OTU) that were possibly contaminated were taxonomically classified against the National Center for Biotechnology Information (NCBI) database using blastn [74]. OTUs whose taxonomy did not match the phylogenetic position in the tree were classified as contaminated sequences and discarded. Sequences in the alignment were then clustered with Clustering 16S rRNA for OTU Prediction (CROP) using the parameters -l 3 and–u 4 [75]. Representative sequences of each OTU were blasted in NCBI blastn database Nucleotide collection nr/nt [74]. For OTU reference sequences with identity values higher than 98% and full query cover the top blast hit was assigned as the taxonomy to the OTU. Assigned taxonomies through blastn were compared with morphological identifications done in the field. Where possible, specimens that were not assigned taxonomy based on their MT-CO1 sequence were allotted a taxonomy using the morphological identification. Those that after a morphological inspection were not able to be assigned to a specific OTU were considered “not assigned” and excluded from the analysis. An OTU table was created in mothur with the files obtained from CROP [76]. Sequences were deposited in the Barcode of Life Data System database with DOI http://dx.doi.org/10.5883/DS-ARMS1617.

### Data analysis

Although we used a molecular and visual identification approach, we were unable to assign all the organisms to an OTU. To minimize biases in the statistical analysis of the data three different subsets of the data were used. 1) To minimize sampling bias, we only assessed ARMS that had at least 60% of the organism collected assigned to a taxonomically classified OTU. Using this threshold resulted in a total of 39 ARMS being retained (S1 File). This subset of 39 ARMS was used for the multivariate tests, species accumulation analysis and rarefaction curves (S1 File) For the univariate analyses and Venn Diagrams, a smaller subset of nine ARMS per region were randomly selected using JMP Pro 15 [77] to reduce biases due to unequal sampling per region. 3) A final subset of the data was used for the expected number of species in 20 individuals with eight randomly selected ARMS being assessed in each region (S1 File). The use of eight ARMS per region was due to the fact that the northern region only having eight ARMS which contained at least 20 organisms.

Chla concentrations, particulate organic carbon (POC), PAR, and SST as monthly averages were obtained from the NASA Goddard Space Flight Center, Ocean Ecology Laboratory, Ocean Biology Processing Group. Moderate-resolution Imaging Spectroradiometer (MODIS) Aqua satellite system at NASA OB.DAAC, Greenbelt, MD, USA with a 4x4 km resolution for each location using the package obpgcrawler R [78], accessed on 02/19/2020. Chla concentrations were obtained from the dataset AQUA MODIS Level-3 Mapped Chlorophyll, Version 2018; doi: 10.5067/AQUA/MODIS/L3M/CHL/2018. POC was obtained from the dataset AQUA MODIS Level-3 Mapped Particulate Organic Carbon, Version 2018; doi: 10.5067/AQUA/MODIS/L3M/POC/2018. PAR was obtained from the dataset AQUA MODIS Level-3 Mapped Photosynthetically Available Radiation, Version 2018; doi: 10.5067/AQUA/MODIS/L3M/PAR/2018. SST was obtained from the dataset AQUA MODIS Level-3 Mapped 11μm Day/Night Sea Surface Temperature, Version 2014; doi: 10.5067/AQUA/MODIS/L3M/SST/2014. Based on the data obtained, we generated the following explanatory variables for each reef to be used in downstream analysis:

i) Annual average over 2013–2017 period of SST, Chla, POC, PAR;

ii) Average over 2013–2017 and the deployment period of the SST differences between hottest and coldest month;

iii) Average over the deployment period of SST (Dep.SST), Chla (Dep.Chla), POC (Dep.POC), and PAR (Dep.PAR).

Initially, we tested the correlation between explanatory variables using the Pearson correlation [79]. Values higher than |0.9| in the Pearson correlation coefficient were considered highly correlated, and only one variable was chosen as a proxy for all. The correlation of explanatory variables showed that satellite-derived variables measured during the deployment period and for 5 years were highly correlated. POC and Chla were also highly correlated. The temperature variation measured as the SST differences between the hottest and coldest month was negatively correlated with SST. We kept for further analysis the 5 years average. In the end, we analyzed the following potential explanatory variables: SST, Chla, PAR, and percentage coverage of SC, HC, Turf, MA, and Abiotic benthic categories (S1 File). To observe differences in the non-correlated environmental variables between regions we performed a Kruskal-Wallis test between regions for each non-correlated environmental variable.

Relationships in the number of species or abundance and regions was investigated. One-Way Analysis of Variance (ANOVA) was used when the assumptions of normally distributed data and homogeneity of variance were met. The non-parametric test Kruskal-Wallis was used when One-Way ANOVA assumptions were not met. The process was repeated for the most abundant phyla (Annelida, Arthropoda, Echinodermata, Mollusca, and Chordata). These phyla combined accounted for 95% of the OTUs and individuals, and individuals of these phyla occurred in at least 40% of the ARMS in each region. Hurlbert’s expected number of species in 20 individuals (ES_20_) was used to standardize the density of individuals in each ARMS. ES_20_ was calculated for each region in R using the package vegan [80].

Species accumulation and rarefaction curves were computed in R using the package vegan to assess if our sampling effort was representative of the cryptic community [81]. A Venn diagram was done in mothur to visualize the number of exclusive and shared species in and between regions [76]. An additional Venn diagram was done to visually observe the number of shared OTUs between the central region, the Al Lith reefs S1, S2, and S3, and the Farasan Island reefs S4 and S5.

A distance-based redundancy analysis (dbRDA) based on the dissimilarity matrix generated with the Bray-Curtis index was performed in the package vegan for R to investigate possible relationships between each of the selected environmental variables and the cryptobiome community structure [81]. Environmental variables were normalized prior to analysis. A permutation test was performed in vegan to test for significance in the dbRDA model [82], using 999 number of permutations.

A similarity percentage analysis (Simper) was performed in vegan for R with 999 number of permutations to investigate the contribution of each OTU to the overall Bray-Curtis dissimilarity between regions. To investigate the relationship between reef sites and the taxa influencing the most the Bray-Curtis dissimilarity patterns between regions, as indicated by the Simper analysis, a second dbRDA was performed using those OTUs.

Classification and regression tree (CART) models were used to investigate responses of the cryptobiome to environmental drivers [83] using SPSS (Version 25). CART models are decision tree models that enable nonlinear relationships and interactions to be analyzed. Models were conducted on the total number of OTUs per ARMS as well as the cryptic organisms that contributed the most to the dissimilarity between regions. Trees explain variation in a single response variable by repeatedly splitting the data into two more homogeneous groups, using the explanatory variable with the highest statistical association to the response variable [84]. A node represents a single input variable and a split point on that variable. The tree is built by splitting the data, constituting the root node of the tree, into subsets (i.e., children nodes also known as second, third etc. nodes). Tree growth was constrained to have a minimum of six observations in a node before attempting a split. As regression tree analysis does not have any distributional assumptions, no transformations were applied.

## Results

### Environmental variability

Five-year mean SST lowest values (26.9°C) were observed in Duba and highest (29.7°C) in the southern reefs (S1 File), which aligns with the predicted environmental gradient known in the Red Sea. However, the temperature difference between the mean of the hottest and the mean of the coldest months lowest values were obtained in the southern reefs (4.5°C) and highest in Duba (7.5°C). Chla concentration also varied among regions, with the southern region, especially at the Farasan Islands, having the highest values (1.74 to 2.52 mg m^-3^, Fig 1 and S1 File). Significant differences were found between regions in all non-correlated satellite-derived environmental variables (S1 File). Regarding the biological and abiotic data resulting from the benthic reef surveys, no significant differences were detected among regions, except for macroalgae (ChiSquare = 7.9, p = 0.02) probably due to high variability in all benthic variables between reefs within the same region (S1 File).

### Biodiversity patterns

#### Regional alpha diversity

**Fig 2:**
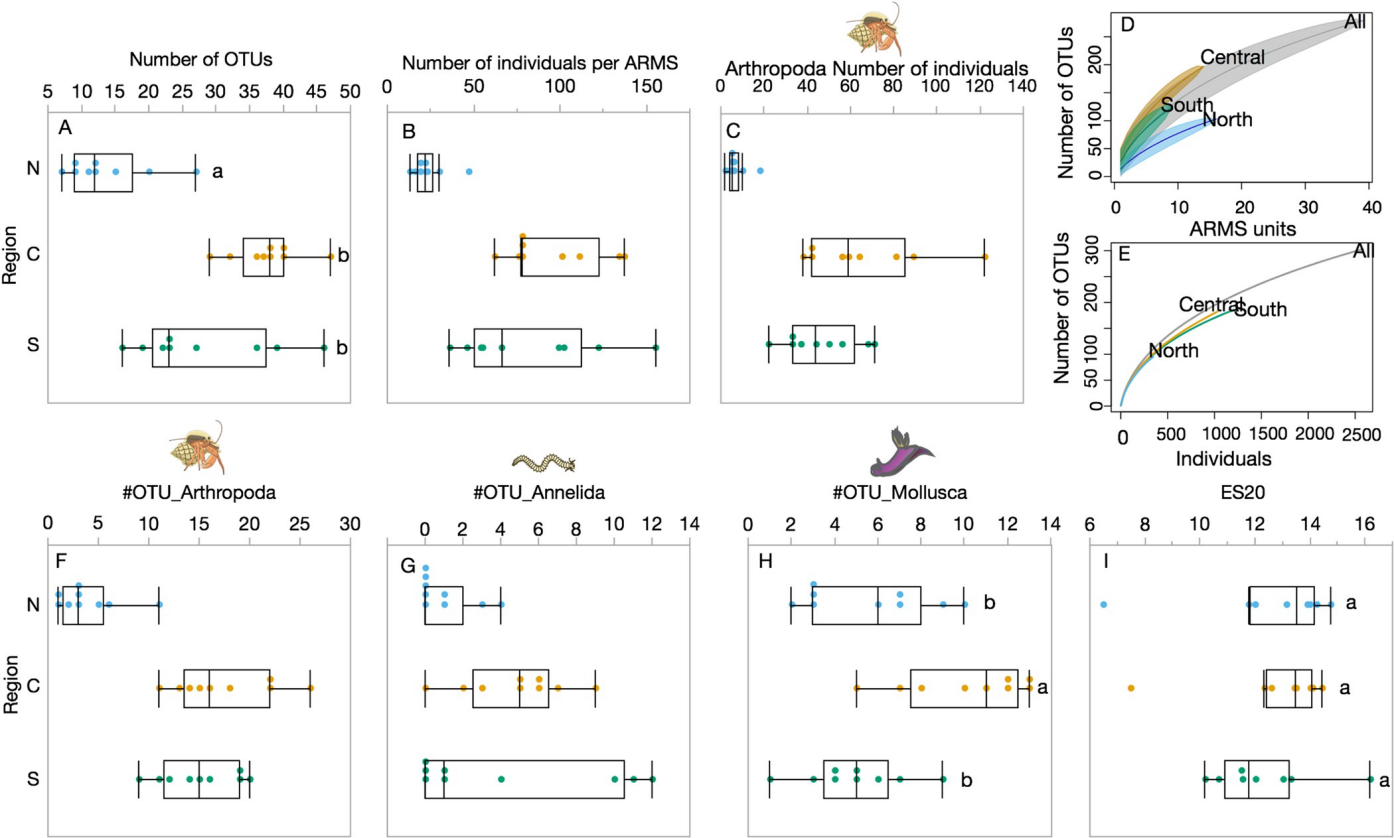
Alpha diversity matrix in the Red Sea basin. A) Boxplot with confidence interval of the mean number of operational taxonomic units (OTUs) per region. B) Boxplot with confidence interval of the mean number of organisms per region. C) Means and confidence intervals of the number of individuals of Arthropoda per region. D) OTUs accumulation curves per ARMS for each region (North, Central, and South) and all ARMS pooled together (All). E) Rarefaction curves per region (north, central, and south) and with all samples pooled together. F) Means and confidence intervals of the number of OTUs per region visualized as a box plot for Arthropoda, G) for Annelida, and H) for Mollusca. I) The expected number of species in 20 individuals [ES20]. The letters a and b denote groups significantly different tested by Tukey HSD test. Data in figures A, H, and I were normally distributed and with equal variances, therefore a One-Way ANOVA was performed. Figures B, C, F, and G were analyzed using Kruskal-Wallis as a not parametric test. ARMS used in each analysis are noted in the S1 File. Images from: Sander Scheffers (hermit crab), Dieter Tracey (Polychaeta), and Tracey Saxby (goby and nudibranch), IAN Image Library (ian.umces.edu/imagelibrary).

From the 2583 successfully obtained MT-CO1 sequences from 54 ARMS recovered along the latitudinal gradient, a total of 279 OTUs were identified. The univariate tests showed that the northern Red Sea region had a significantly lower average number of OTUs (F = 23.14, p = 0.0001) compared to the central and southern regions, which were not significantly different (Fig 2A) from each other. The averages in the number of individuals were significantly different between regions (ChiSquare = 17.18, p = 0.0002; Fig 2B). Rarefaction curves did not reach a plateau when plotting all the sampling units together, suggesting that biodiversity was underestimated (Fig 2D). The central Red Sea had a slightly higher accumulation of OTUs per ARMS and per number of individuals sampled (Fig 2D and 2E). The ES_20_ value was very variable per reef, but similar amongst regions (Fig 2I).

Annelida, Arthropoda, and Mollusca showed significant differences in the number of OTUs among regions (Fig 2F–2H). The mean number of OTUs of Annelida (1.0 OTUs per ARMS in the north, 4.8 in the central, and 4.3 in the south; ChiSquare = 6.4, p = 0.04), Arthropoda (3.8 OTUs per ARMS in the north, 17.4 in the central, and 15.0 in the south; ChiSquare = 17.01, p < 0.001), and Mollusca (5.5 OTU per ARMS in the north, 10.1 in the central, and 4.9 in the south; F = 9.91, p < 0.001) and the abundance of Arthropoda (6.5 individuals per ARMS in the north, 65.9 in the central, and 46.0 in the south; ChiSquare = 18.67, p < 0.001) were different between regions (Fig 2C, 2F–2H). The mean number of OTUs of Arthropoda and Annelida, and the mean number of individuals of Arthropoda was lower in the northern Red Sea.

#### Distribution of the number of OTUs between regions

**Fig 3:**
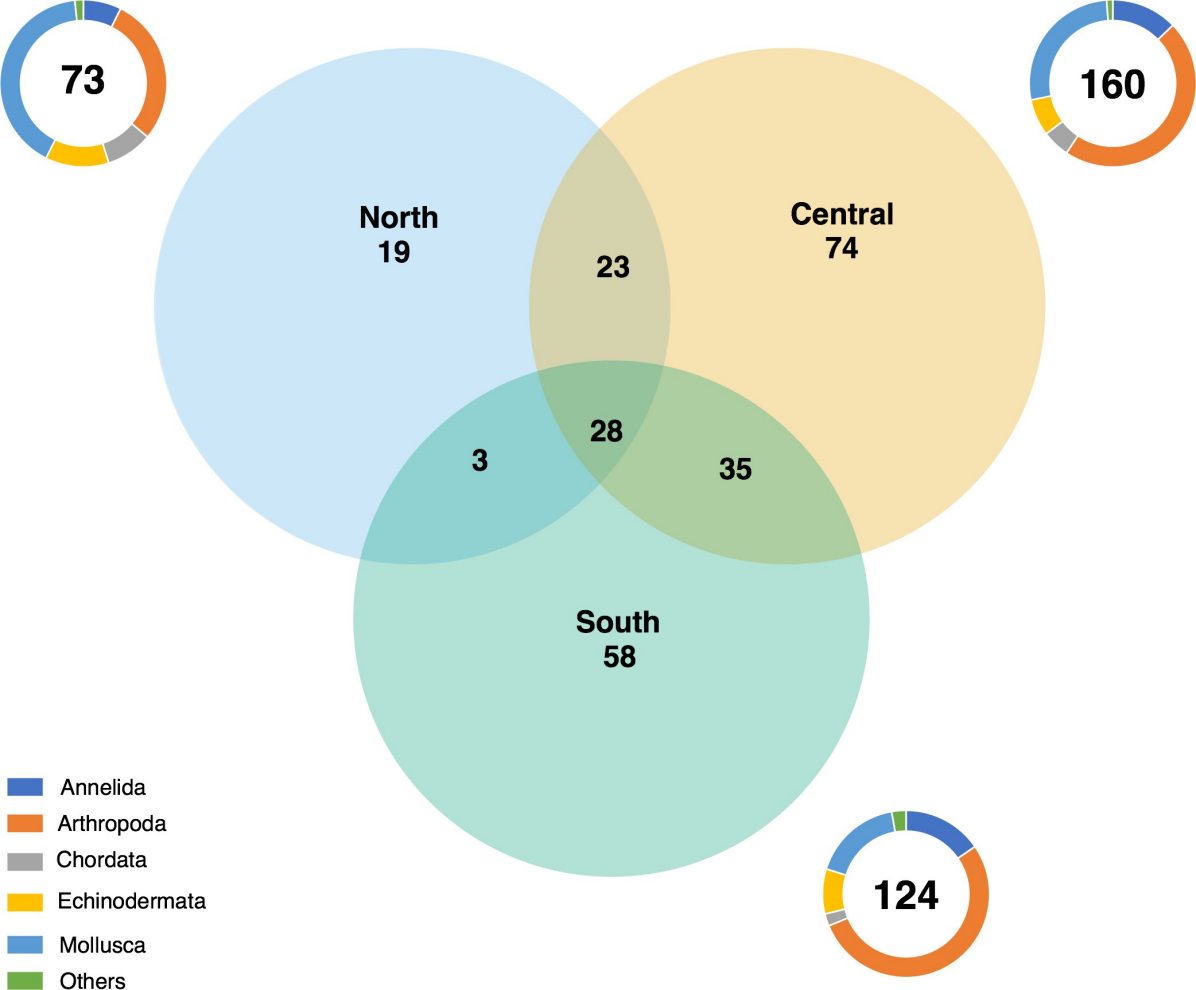
Venn diagram showing the number of operational taxonomic units (OTUs) unique to each region and shared between the regions (North, Central, and South) using a random subsample of nine ARMS per region. The associated circle graphs show the relative composition of cryptobiome at the Phylum level calculated in terms of the relative number of OTUs per region (numbers shown in the center of each circle). The phyla Platyhelminthes, Nemertea, and Sipuncula were grouped under the category “others”.

Approximately 63% (151 OTUs) of the OTUs were exclusive to a single region, 25% (61 OTUs) were shared between two regions, and 13.8% (28 OTUs) were present in all regions. Among the shared OTUs, 35 OTUs (15% of the total OTUs) were shared between the central and southern regions of the Red Sea. Also, 10% (23 OTUs) of the OTUs were shared between the central and northern regions, and 1% (3 OTUs) between the northern and the southern regions (Fig 3). The central region shared 12% of the central and south OTUs (30 OTUs) with S1, S2, and S3 reefs located in Al Lith and 7% of the central and south OTUs (17 OTUs) with S4 and S5 located in the Farasan Islands. The phyla Annelida, Arthropoda, and Mollusca accounted for the most abundant OTUs in the central and southern regions (Fig 3). Arthropoda, Echinodermata, and Mollusca accounted for the majority in the northern region. Arthropoda was the phylum with the largest number of OTUs in the central and south regions, while Mollusca dominated in the north.

#### Linking environmental variables to community patterns

**Fig 4:**
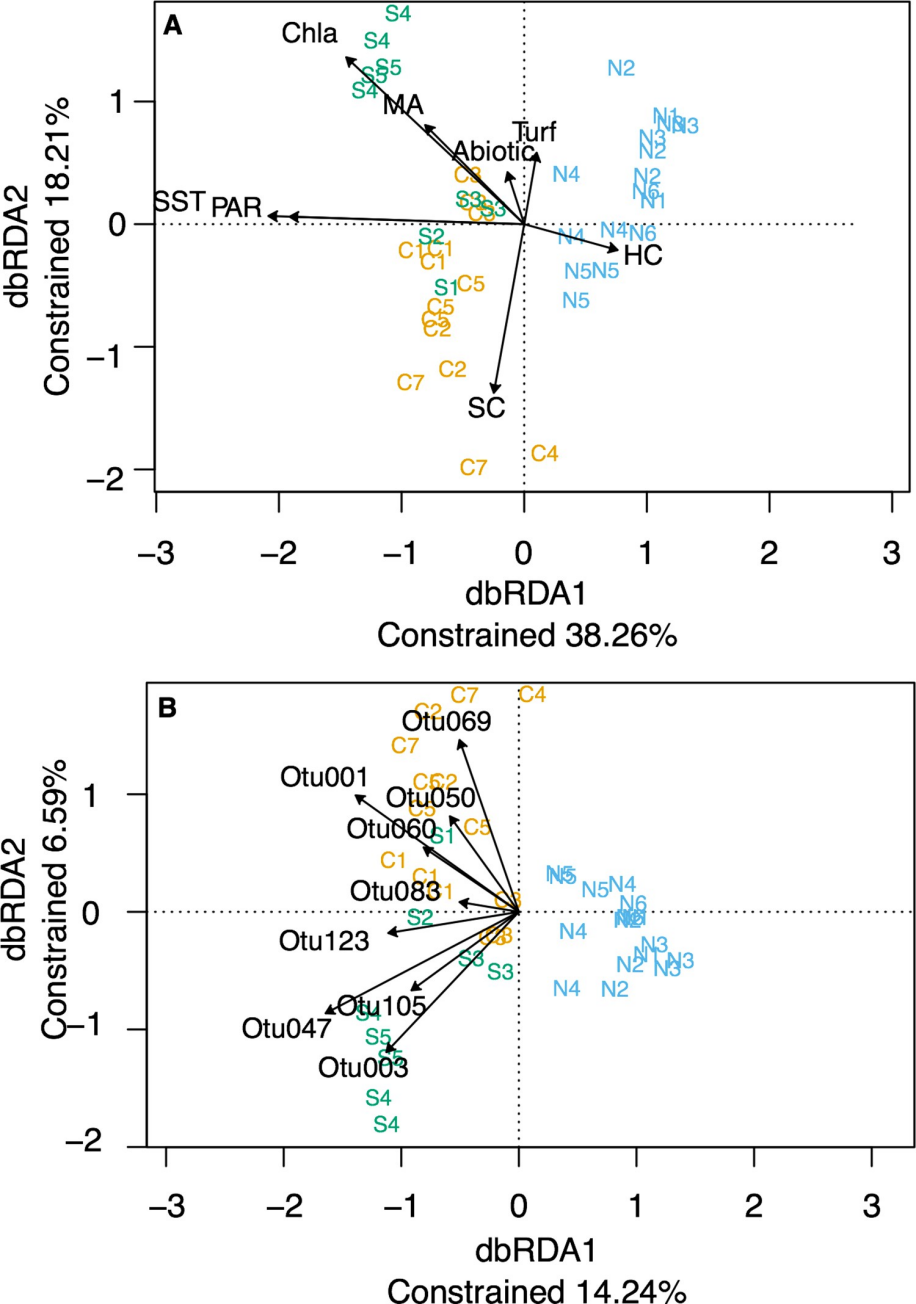
Multivariate visualizations of the distance-based redundancy analysis (dbRDA) showing differences between the community structure of cryptic organisms of the ARMS sampled in the north (N), central (C), and south (S) regions. The axis represents the percentage of constrained variation. A) The arrows mark the contribution of the non-correlated explanatory variables to the variation observed. Sea surface temperature (SST), Chlorophyll-a concentration (Chla), and photosynthetic active radiation (PAR) were obtained from remote sensing and calculated as averages of the monthly means over five years prior to ARMS retrieval dates. Reef community variables from reef surveys at 10 m depths as in percentage cover of hard corals (HC), percentage cover of macroalgae (MA), percentage cover of soft coral (SC), percentage cover of turf algae (Turf), percentage cover of bare substrates (Abiotic; i.e., rock, sand, and dead coral) were also correlated with observed cryptic biodiversity patterns. B) The arrows mark the contribution of the OTUs that significantly aided to the differences obtained in community structure between two regions.

In the distance-based redundancy analysis, PAR and SST are mostly associated with the first axis that explained 38.26% of the constrained variation (Fig 4A). Percentage cover of soft corals was associated with the second axis that explained 18.21% of the constrained variation and separated central from southern reefs with exception of S2 and S1. Soft corals were associated with most of the assemblages of the reefs in the central region. The northern reefs were associated with percentage cover of hard corals. Turf algae was associated with the second axis. The southern reefs and C3 were associated in the top left quadrat with Chla and macroalgae and at a lesser extent with the abiotic variables. The permutational multivariate analysis of variance showed that the dbRDA results are not random (p = 0.001).

The dissimilarity in the communities between regions was driven in its majority by OTUs of Arthropoda based on the results from the Simper analysis (S1 File). The Palaeomonidae shrimps OTUs 123 and 47, the crab *Tanaocheles stenochilus* OTU 105, and the brittle star *Ophiotrix* OTU 3 were associated with both the first and the second axis in the bottom left quadrant. This quadrant had ARMS from all the southern reefs present and two ARMS from C3. The Paguridea OTU 1, OTU 83, and OTU 50, the decapod OTU 60, and the Galatheidae OTU 69 were associated with both axes in the top left quadrant with the ARMS from the central Red Sea except one ARMS from C4 and two form C2. All northern Red Sea reefs clustered to the right separated by the first axis from the south and central Red Sea reefs. No OTU used in this analysis was associated with the north Red Sea reefs.

#### Environmental variables influence the cryptobiome distribution patterns

Classification and Regression Tree models were used to investigate important environmental drivers and split points of cryptic diversity. For the nine OTUs that significantly contributed to the dissimilarities observed, SST and PAR were the physico-chemical environmental variables most likely to form the first (parent node) and second split (Table 1). The characteristics of the nearby benthic community can also affect the distribution patterns of cryptic fauna. Hard corals and turf were identified as the most influential coral reef benthic categories and important drivers within the first three nodes of a tree followed by abiotic substrate (Table 1).

**Table 1:** Variables driving the parent and two first nodes (split points) in the regression trees summed overall response variables. Figures in the supporting information shows the complete tree for each variable. Turf, HC and Abiotic stands for means percentage cover of turf algae, hard coral, and not live substrate respectively. Average SST (sea surface temperature) and PAR (photosynthetic active radiation) are satellite-derived variables calculated over a period of 5 years.

	SST	PAR	Turf	HC	Abiotic
Node 1 (Parent)	1	2	3		1
Node 2	3	1		1	
Node 3	1			1	
*Total*	*5*	*3*	*3*	*2*	*1*

**Fig 5:**
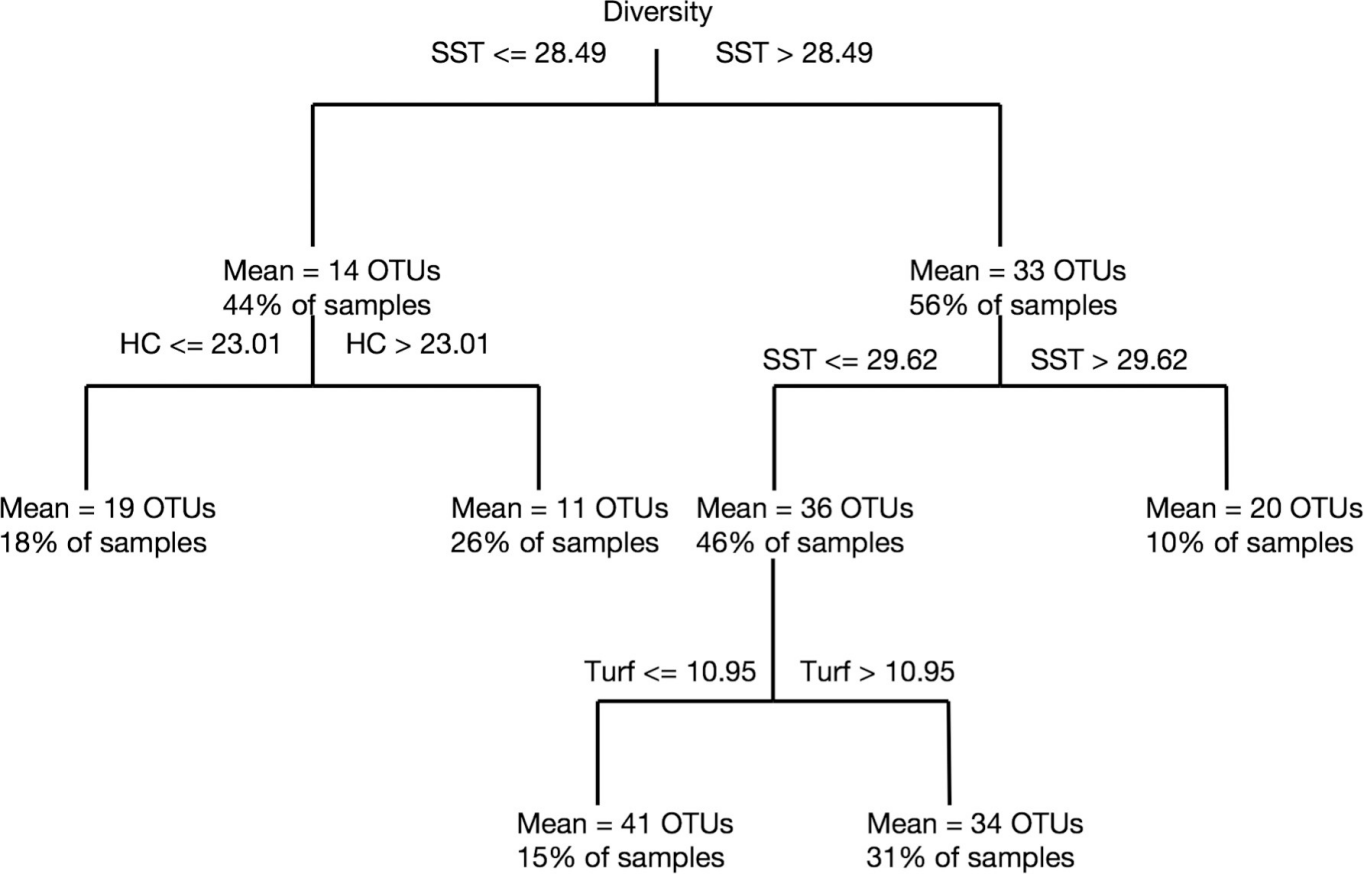
CART model using regression tree to observe critical breakpoints in the OTU numbers as a proxy for species richness of the cryptobiome in response to SST, Chla (and its correlated variable POC), PAR, HC, SC, Turf, and MA.

Overall, cryptic benthic diversity (i.e. number of OTUs) was affected by several environmental drivers including SST, HC, and Turf (Fig 5). SST provided the first-level split for overall diversity where higher SST levels were associated with higher diversity (Fig 5). A lower percentage of hard coral cover and turf cover were associated with higher cryptic diversity, which may be indicative of the overall higher cryptic diversity observed in the central and southern areas. In general SST values of 28.5°C and turf values of less than 41% cover represented critical values where the abundance of common OTUs changed (Table 2). Notably, critical values greater than 28.5°C SST were associated with higher overall diversity of cryptic fauna and two shrimps (OTU 47 and OTU 123). PAR values of greater than 49 Einstein m^-2^ d^-1^ resulted in an increased abundance of the brittle star (OTU 3) and the shrimp (OTU 47). The full tree structures are provided in the S1 File.

**Table 2:** Summary of the key environmental variables and split values identified for which the abundance of each OTU were highest and lowest. Percentage cover of turf algae (Turf), not living substrates (Abiotic), and hard coral (HC), and the five-year average of the remote sensing variables sea surface temperature (SST), and photosynthetic active radiation (PAR) were the key environmental variables determining split values of the dominant OTU abundance.

	Highest Abundance	Lowest Abundance
Diversity	SST > 28.49	SST < 28.49
	HC < 23.01	HC > 23.01
*Exoclimenella* OTU 123	Turf > 41.78	Turf < 41.78
	SST > 29.62	SST < 29.62
Galatheidae OTU 69	Abiotic < 25.26	Abiotic > 25.26
	SST < 29.24	SST > 29.24
*Palaemonella pottsi* OTU 47	PAR > 49.01	PAR < 49.01
	SST > 28.49	SST < 28.49
Paguridae OTU 83	Turf > 34.97	Turf < 34.97
Paguridae OTU 50	Turf > 41.78	Turf < 41.78
	Abiotic < 25.26	Abiotic > 25.26
Paguridae OTU 1	SST > 28.49	SST < 28.49
	PAR > 47.2	PAR < 47.2
*Ophiothrix* OTU 3	PAR > 49.01	PAR < 49.01
	SST > 29	SST < 29
Decapoda OTU 60	Turf > 41.78	Turf < 41.78
	HC > 37.08	HC < 37.08
*Phylladiorhynchus* OTU 78	PAR > 48.38	PAR < 48.38
	SC > 5.45	SC < 5.45
*Synalpheus* OTU 80	Turf > 34.97	Turf < 34.97
	HC < 4.85	HC > 4.85
*Tanaocheles stenochilus* OTU 105	PAR > 49.02	PAR < 49.02

## Discussion

Currently, there is an urgent need to investigate biodiversity patterns and distribution of species in increasingly disturbed ecosystems, such as coral reefs [85]. Understanding the stressors and environmental factors influencing coral reef functioning is particularly useful for marine spatial planning and conservation [86]. Considering the accelerating degradation of coral reefs as a result of global warming, resulting in more frequent, intense, and long lasting bleaching events, the use of standardized approaches and tools has been increasingly advocated to maximize the ability to predict trajectories of change globally and through time [57]. The present study is one of the first to provide information for a better understanding of the responses of the cryptobiome to natural environmental gradients at the scale of a sea basin using standardized tools (ARMS) and quantitative approaches. The sampling locations along these gradients allowed us to test four hypotheses: 1) Temperature will have a positive relationship with species richness; 2) Temperature will affect species composition of the cryptobiome; 3) Energy input shapes species richness and abundance; and 4) Benthic characteristics of the reef habitats influence the cryptobiome.

Most of the OTUs retrieved in this study were exclusive to one region, and 43% of the OTUs were only represented by a single individual. We also found a high variability between reefs in the same region. In part, this variability could be due to the limited sampling area (0.86 m^2^) of ARMS as well as the fact that ARMS in general target species that live in and on the benthic habitat. For example, the genus *Pseudanthias* usually aggregates in schools above coral heads [87] and thus it may be missed from collection although occasionally may be collected in high abundance from an ARMS. However, we believe that the records of high variability in diversity over short distances in the literature and in our results, may reflect the high degree of ecological specialization that exists at coral reefs, probably resulting from the wide range of ecological niches available [88, 89].

As in many other marine biodiversity studies, we could not get an asymptote in the rarefaction and species accumulation curves, suggesting that many of the rare species with the capacity to inhabit the ARMS are yet to be sampled. Therefore, the percentage of rare species in our sampling and the regional biodiversity, is likely underestimated. These species with locally low abundance can provide unique functions in the ecosystem [90] and are more susceptible to local extinction because of its small population size [91]. The focus in maintaining species diversity and protecting rare species becomes relevant for the conservation of coral reefs ecosystem function [92]. Yet, our knowledge of coral reef biodiversity is lacking with the majority of the species in the coral reef still undescribed [1]. Also, there is a need to increase the taxonomically identified genetic barcodes in public databases. For example, in the cryptobiome most of the sequences retrieved with molecular techniques do not have a species level taxonomic match in the public genetic databases [55]. Our current work, contributed to decrease this gap, providing more than 2500 sequences that can match a taxon in the public NCBI database. However, species level identifications in genetic databases are still needed, making the efforts of expert taxonomists important to maximize the advantages of applying molecular tools to describe biodiversity patterns over time and space.

Here, we disentangled the distribution patterns of key players of the cryptobiome as well as of the community structure, in areas along the Red Sea spanning yearly average temperatures from ~27°C to 30°C. The speed of energy transfer in ectotherms is modulated by temperature [35, 93]. This critical variable can also shape communities by favoring species with optimal performance at the ambient temperature and therefore regulating the distribution of species in, for example, coral reefs [17]. Species accumulation and rarefaction curves showed that the colder northern reefs were comparatively poor in the number of OTUs and the number of organisms compared to the warmer central and southern regions. Nevertheless, this difference was attenuated when investigating ES_20_ values, suggesting that the difference in number of OTUs could be a reflection of the number of organisms sampled. Univariate alpha-diversity metrics separated northern reefs from the south and central reefs (i.e., number of OTUs and abundance). The same pattern was also reflected by the number of OTUs of Arthropoda and Echinoderms. However, the similarity in the expected number of OTUs in 20 individuals between regions suggests that the lower number of OTUs in the north is related to the low density of individuals in this region, and more ARMS per reef might be needed to better capture the region’s biodiversity. The shift in dominance from arthropods to mollusks and from annelids to echinoderms, as well as the increase in proportion of mollusks and chordates from south to north further supports the distinction in fauna between regions. Our multivariate analysis showed that five-year average sea surface temperature was associated with the separation of the northern reefs from the south and central reefs both regarding community structure and composition. This is in contrast to Brandl et al., 2020 [94] who reported less diversity of crypto benthic fishes in the warmer Arabian Gulf compared to the colder Gulf of Oman. However, diversity might be restricted by high fluctuations in temperature. Brandl et al., 2020 [94] registered that in the Arabian Gulf temperature can reach 36°C in summer and 17°C in winter and in our multivariate analysis temperature variability between the hottest and coldest months was negatively correlated with five-year average temperature and therefore associated with the distinctions in community structure and composition between regions. The number of shared OTUs between regions supported the distinction in communities between the north and the central and south. However, closer geographical distance between the central region and the southern region reefs S1, S2, and S3 could have masked the distinction between the central and the warmer south region. Indeed, S4 and S5 reefs located in the Farasan Islands shared fewer OTUs with the central region than S1, S2, and S3. The Farasan Islands have a unique geomorphology and are directly influenced by water masses originating in the Indian Ocean [65] and previous studies with ARMS targeting bacteria have shown an uniqueness of associated communities in the region [68]. Future studies increasing the sampling size in the Farasan Islands will allow analyzing them as a separate region to unveil the distinct community that might be present. Species inhabiting Red Sea reefs could be readily acclimated or adapted to local temperatures [95, 96], however the effects of temperature could still influence their metabolic rate. For example, temperature is an important limiting factor for the distribution of types of *Symbiodinium* from the host *Palythoa tuberculosa* in the Red Sea. Congruent with our results, SST may affect community structure [97] and food web dynamics in marine ecosystems [98], however continuous warming can also have negative impacts on reef biodiversity. Indeed, modifying the species distributions at lower trophic levels may have repercussions that will be extended through the food chain [5, 8], affecting higher trophic levels like larger fish communities. The implications of the continuous warming in the oceans, and in particular in coral reefs, can have deleterious impacts on reef biodiversity, considering this is dominated by the cryptobiome. Indeed, from the hundreds of OTUs collected, varying temperature optima are expected. The CART models showed, for example, that the abundance of the common shrimps (OTU 47 and OTU 123) and a squat lobster (OTU 69) were associated with increasing temperature, displaying higher abundance in areas with temperature above 28°C. The northern Red Sea is projected to increase by 0.4°C per decade, challenging the persistence of species adapted to colder temperatures in this region [99]. This change might result in distribution shifts of warm adapted species, such as the common shrimps and squat lobster mentioned above, towards the north [99], and ultimately in the hominization of the reef biodiversity in the region. On a global scale, there is evidence that temperature positively influences species richness in several terrestrial and marine taxa [93, 100]. However, the lack of a sound knowledge on the ecological patterns of most of these species limits our ability to confidently conclude future trajectories in the Red Sea coral reefs under climate change scenarios. Mesocosm experiments should be performed to better understand and forecast their responses to single and combined stressors.

We observed that PAR and Chla concentration (and POC due to its high correlation with Chla) influenced differences in the community composition of the Red Sea cryptobiome. Indeed, communities with higher access to energy, such as areas with high biomass of primary producers, can have a distinct structure compared to areas with less access to energy [35]. One possible mechanism for the change in community composition occurs when species with higher tolerance to increased yields of primary producers outcompete the current dominant species [101]. Our CART models using regression tree analysis also showed that the availability of energy as PAR was an important factor associated with abundance for certain taxa. The absorption of PAR by autotrophic communities and associated fixation of carbon crucially underpins energy transfer in almost all ecosystems including marine environments [102]. Previous ecosystem function studies demonstrate a positive relationship between plant diversity and primary productivity [103]. It is therefore not surprising that higher light levels supported overall higher cryptic diversity likely as a consequence of increased primary productivity. The northern Red Sea reefs surveyed, where average PAR values are generally lower compared to the rest of the basin, showed significantly lower abundance of organisms and species than those in the central and south Red Sea. The hermit crab Paguridae OTU 1, the brittle star *Ophiothrix* OTU 3, the shrimp *Palaemonella pottsi* OTU 47, and the crab *Tanaocheles stenochilus* OTU 105 all showed an increased abundance with PAR values above 48 or 49 Einstein m^-2^ d^-1^. However, our dataset only contained a single reef above this threshold and further investigations with a larger gradient of PAR values would be required to give additional information on the effects of PAR to the cryptobiome. Additionally, experimental work can also help to obtain a better understanding of the response of species to single and multiple variables relevant for species metabolism.

Our results showed that the amount of energy available that enters the trophic web is associated with species diversity and abundance in the cryptobiome. Therefore, changes in the natural background levels of energy available in the system, resulting among others from eutrophication of coastal areas in oligotrophic seas will cause shifts on the cryptobiome biodiversity, in line with what has been found for larger coral reef communities [104]. The energy available for some organisms in oligotrophic waters like the Red Sea is generally scarce [69]. Indeed, corals rely on benthic dinitrogen fixation [105] and the recycling of carbon and nitrogen [106]. An imbalance from an intensive intrusion of nutrients in coral reefs can cause benthic algae to outcompete corals [107]. In nitrogen rich environments, corals have smaller temperature thresholds to bleaching [108]. Therefore, energy available is likely a limiting factor for the distribution of some species. Species in the lower trophic levels of the cryptobiome might be more vulnerable to changes in the energy available from primary producers and cascade effects might happen, which can ultimately result in the shift of the reef cryptobiome.

Coral reefs present a variety of niches that harbor distinct mobile animal communities during parts of their life cycle [88, 89]. Different reef benthic categories (e.g., hard corals, soft corals, turf, rubble) can harbor distinct assemblages of species [41]. We hypothesized that the characteristics of the nearby reef habitat would drive community composition differences in the cryptofauna. Our results showed that the regions differed in the environmental characteristics of the water column but not on most of the benthic coral reef communities assessed by photo transects, probably due to high variability among transects in the same reef. Nevertheless, the multivariate analysis (dbRDA) showed an influence of major benthic categories shaping the ecological patterns of the Red Sea cryptobiome. In particular, the percentage cover of turf algae and hard corals played an important role on species diversity patterns. Indeed, turf algae harbors high diverse and abundant assemblages [109] and hard corals shelter unique parasitic, commensal, and symbiotic associated cryptic fauna [47, 110, 111]. We found a threshold of 23% of coral cover and 11% of turf algae cover for the cryptobiome diversity, with higher number of OTUs below these thresholds. These categories that characterize the nearby habitats and that can harbor a great part of the pool of ARMS colonizers were also determinant for individual species. For example, the abundance of the pagurid crabs OTU 83 and OTU 50, and the shrimps *Exoclimenella* OTU 123 and *Synalpheus* OTU 80 were primarily affected by the percentage cover of turf algae. Our dataset included only one reef above the threshold of percentage cover of turf algae for *Exoclimenella* OTU 123 and Paguridae OTU 50. A larger dataset is recommended to further explore the effect of turf algae in these OTUs. Multiple examples of substrate preference by specific coral reef dwellers have been documented [41]. For example, *Trapezia* and *Tetralia* seemed as obligate dwellers of *Pocillopor*a and *Acropora* respectively [112], but most likely it is the structure provided by the coral colony itself that play a major role, as they use dead coral colonies in part of their life cycles [113]. We provide further evidence of the importance of benthic substrates for coral reef communities [50]. With global warming, coral reef benthic communities are expected to shift towards a dominance of algae due to their comparatively higher resistance to temperature increase compared to corals [114, 115]. This shift could influence crytobenthic fishes as well, which are strongly influenced by the presence of branching corals [97]. The shift in fish communities with decreasing coral cover [116] could cascade through predation to lower trophic levels in the cryptobiome [8], possibly explaining the decrease in diversity observed at a higher percentage of coral cover. Given the dependence of the cryptic species on the prevalent benthic categories in a certain reef, the anticipated shifts due to global warming will also be reflected at the micro biological scales, even though these will remain overlooked with traditional monitoring (e.g., line-intercept or photo-transect methods).

## Conclusions

We showed that the largest size fraction of the cryptobiome (>2000 μm) is characterized by a high number of species with low abundances, as reported in previous studies of the cryptobiome. The uniqueness of the cryptobiome communities across regional scales stresses the need for a balanced distribution of conservation efforts (e.g., marine protection areas) along the entire Saudi Arabian Red Sea coastline to protect the largest levels of biodiversity possible. We found that i) the number of species would increase with higher temperature; ii) the community composition changed along the temperature gradient; iii) the number of species and abundance would increase with higher energy input; iv) the characteristics of the nearby reef benthos to influence the cryptobiome. Indeed, the influence of percentage cover of hard corals and turf algae on the number of OTUs and the distribution of key organisms suggest that biodiversity of coral reefs cryptobiome may shift in the future in response to changes in the macro- and megabenthic communities. The negative influence of hard coral cover and the positive influence of temperature on the cryptobiome diversity suggests a local increase in diversity of the cryptobiome with future predictions of temperature rise and increasing recurrence of bleaching events. However, considering the very limited knowledge that exists on this component of the reef biodiversity, under scenarios of reef degradation due to local and global pressures, we can also assist to a homogenization of the cryptobiome, if the warm-adapted species of the central and south Red Sea could outcompete the cold adapted species in the north areas. Molecular based techniques in combination with standardized tools can be valuable to characterize the baseline biodiversity and assess trajectories of change even if species cannot have a proper taxonomic identification.

## Supporting information

S1 File Tables and figures in the supporting information include. Metadata for each ARMS retrieved. Pearson correlation between explanatory variables obtained from remote sensing data and benthic surveys. Significance values of the differences of satellite derived products and percentage cover of ecologically meaningful benthic structures between regions. The 10 highest averages of the OTUs influencing differences in community structure obtained using a Simper analysis with 999 permutations and their respective taxonomy. Venn diagram showing the number of operational taxonomic units (OTUs) unique to the central region and Al Lith (S1, S2, and S3) and Farasan Islands reefs (S4 and S5) and shared between them using a random subsample of 9 ARMS of the central region. CART models using regression tree to distinguish critical breakpoints in the abundance of the OTUs that significantly contributed to the dissimilarities observed.
(PDF)
