# Supplementary material for: Responses of the coral reef cryptobiome to environmental gradients in the Red Sea
Source: PLoS One. 2024 Apr 16;19(4):e0301837. doi: 10.1371/journal.pone.0301837 (PMC11020721; doi:10.1371/journal.pone.0301837)
Supplement: S1 File — Metadata for each ARMS retrieved. Pearson correlation between explanatory variables obtained from remote sensing data and benthic surveys. Significance values of the differences of satellite derived products and percentage cover of ecologically meaningful benthic structures between regions. The 10 highest averages of the OTUs influencing differences in community structure obtained using a Simper analysis with 999 permutations and their respective taxonomy. Venn diagram showing the number of operational taxonomic units (OTUs) unique to the central region and Al Lith (S1, S2, and S3) and Farasan Islands reefs (S4 and S5) and shared between them using a random subsample of 9 ARMS of the central region. CART models using regression tree to distinguish critical breakpoints in the abundance of the OTUs that significantly contributed to the dissimilarities observed. (PDF) [file pone.0301837.s001.pdf]

Additional Information

Table S1. Metadata for each ARMS retrieved. X marks the analysis in which each ARMS was used. In Region N is north, C central, and S south. Temperature values for sea surface temperature (SST) are in Celsius; chlorophyll – a (Chla) and particulate organic carbon (POC) concentration values in mg m^{-3}; photosynthetically active radiation values are in Einstein m^{-2} d^{-1}. Hard coral (HC), soft coral (SC), turf algae (Turf), macro algae (MA), not living substrate (Abiotic) are values of percentage cover in the benthic surveys.

Reef_code	Reef_number	Region	ES(20)	Boxplots #ind and OTUs	Simper	dbRDA	CART	Deployment	Retrieval	Latitude	Longitude	SST	Dep.SST	Chla	Dep.Chla	POC	Dep.POC	PAR	Dep.PAR	HC	SC	Turf	MA	Abiotic	SSTVar	Dep.SST Var
DR09A	N1	N		X	X	X	X	Aug - 14	Jul - 16	27.936	35.269	26.919	27.000	0.337	0.353	98.983	100.275	47.022	47.082	23.840	2.373	17.476	0.216	43.042	7.618	7.440
DR09B	N1	N		X	X	X	X	Aug - 14	Jul - 16	27.936	35.269	26.919	27.000	0.337	0.353	98.983	100.275	47.022	47.082	23.840	2.373	17.476	0.216	43.042	7.618	7.440
DR09C	N1	N						Aug - 14	Jul - 16	27.936	35.269	26.919	27.000	0.337	0.353	98.983	100.275	47.022	47.082	23.840	2.373	17.476	0.216	43.042	7.618	7.440
DR08A	N2	N			X	X	X	Aug - 14	Jul - 16	27.922	35.232	26.919	27.000	0.337	0.353	49.056	50.408	47.022	47.082	32.910	0.346	20.554	0.346	39.030	7.618	7.440
DR08B	N2	N		X	X	X	X	Aug - 14	Jul - 16	27.922	35.232	26.919	27.000	0.337	0.353	49.056	50.408	47.022	47.082	32.910	0.346	20.554	0.346	39.030	7.618	7.440
DR08C	N2	N			X	X	X	Aug - 14	Jul - 16	27.922	35.232	26.919	27.000	0.337	0.353	49.056	50.408	47.022	47.082	32.910	0.346	20.554	0.346	39.030	7.618	7.440
DR10A	N3	N			X	X	X	Aug - 14	Jul - 16	27.848	35.313	27.025	27.276	0.304	0.318	225.014	197.240	46.475	46.549	30.355	0.802	25.200	0.458	33.677	7.593	7.605
DR10B	N3	N		X	X	X	X	Aug - 14	Jul - 16	27.848	35.313	27.025	27.276	0.304	0.318	225.014	197.240	46.475	46.549	30.355	0.802	25.200	0.458	33.677	7.593	7.605
DR10C	N3	N	X	X	X	X	X	Aug - 14	Jul - 16	27.848	35.313	27.025	27.276	0.304	0.318	225.014	197.240	46.475	46.549	30.355	0.802	25.200	0.458	33.677	7.593	7.605
DR12A	N4	N	X	X	X	X	X	Aug - 14	Jul - 16	27.681	35.440	27.002	27.159	0.194	0.196	101.231	102.183	46.718	46.875	6.032	5.291	28.148	0.000	59.153	7.192	7.160
DR12B	N4	N	X		X	X	X	Aug - 14	Jul - 16	27.681	35.440	27.002	27.159	0.194	0.196	101.231	102.183	46.718	46.875	6.032	5.291	28.148	0.000	59.153	7.192	7.160
DR12C	N4	N	X		X	X	X	Aug - 14	Jul - 16	27.681	35.440	27.002	27.159	0.194	0.196	101.231	102.183	46.718	46.875	6.032	5.291	28.148	0.000	59.153	7.192	7.160
DR07A	N5	N	X		X	X	X	Aug - 14	Jul - 16	27.273	35.626	26.915	27.066	0.166	0.171	52.275	53.342	47.350	47.346	20.043	15.168	30.878	0.758	28.494	6.850	6.882
DR07B	N5	N	X	X	X	X	X	Aug - 14	Jul - 16	27.273	35.626	26.915	27.066	0.166	0.171	52.275	53.342	47.350	47.346	20.043	15.168	30.878	0.758	28.494	6.850	6.882
DR07C	N5	N	X		X	X	X	Aug - 14	Jul - 16	27.273	35.626	26.915	27.066	0.166	0.171	52.275	53.342	47.350	47.346	20.043	15.168	30.878	0.758	28.494	6.850	6.882
DR06A	N6	N						Aug - 14	Jul - 16	27.077	35.787	26.895	27.047	0.179	0.182	97.842	58.550	47.194	47.054	26.344	20.430	8.495	0.650	41.505	6.576	6.797
DR06B	N6	N		X	X	X	X	Aug - 14	Jul - 16	27.077	35.787	26.895	27.047	0.179	0.182	97.842	58.550	47.194	47.054	26.344	20.430	8.495	0.650	41.505	6.576	6.797
DR06C	N6	N	X	X	X	X	X	Aug - 14	Jul - 16	27.077	35.787	26.895	27.047	0.179	0.182	97.842	58.550	47.194	47.054	26.344	20.430	8.495	0.650	41.505	6.576	6.797
ASHAA	C1	C	X		X	X	X	Jun - 15	Jun - 17	22.298	39.044	28.576	28.705	0.296	0.285	91.731	86.771	48.404	48.505	8.724	3.926	44.493	0.000	40.676	5.748	5.922
ASHAB	C1	C		X	X	X	X	Jun - 15	Jun - 17	22.298	39.044	28.576	28.705	0.296	0.285	91.731	86.771	48.404	48.505	8.724	3.926	44.493	0.000	40.676	5.748	5.922
ASHAC	C1	C	X	X	X	X	X	Jun - 15	Jun - 17	22.298	39.044	28.576	28.705	0.296	0.285	91.731	86.771	48.404	48.505	8.724	3.926	44.493	0.000	40.676	5.748	5.922
AFHLA	C2	C						Jun - 15	Jun - 17	22.228	38.965	28.580	28.690	0.701	0.610	162.449	146.571	48.105	48.121	41.253	5.616	17.495	0.108	22.138	5.887	5.965
AFHLB	C2	C		X	X	X	X	Jun - 15	Jun - 17	22.228	38.965	28.580	28.690	0.701	0.610	162.449	146.571	48.105	48.121	41.253	5.616	17.495	0.108	22.138	5.887	5.965
AFHLC	C2	C			X	X	X	Jun - 15	Jun - 17	22.228	38.965	28.580	28.690	0.701	0.610	162.449	146.571	48.105	48.121	41.253	5.616	17.495	0.108	22.138	5.887	5.965
ASHFA	C3	C		X	X	X	X	Jun - 15	Jun - 17	22.139	38.968	28.622	28.700	0.768	0.675	174.575	162.714	48.350	48.471	13.939	4.224	39.071	0.422	38.543	6.025	5.815
ASHFB	C3	C	X		X	X	X	Jun - 15	Jun - 17	22.139	38.968	28.622	28.700	0.768	0.675	174.575	162.714	48.350	48.471	13.939	4.224	39.071	0.422	38.543	6.025	5.815
ASHFC	C3	C	X	X	X	X	X	Jun - 15	Jun - 17	22.139	38.968	28.622	28.700	0.768	0.675	174.575	162.714	48.350	48.471	13.939	4.224	39.071	0.422	38.543	6.025	5.815
AMDFA	C4	C						Jun - 15	Jun - 17	22.089	38.778	28.423	28.501	0.183	0.193	60.787	76.095	48.494	48.592	22.186	4.113	11.905	0.541	9.091	5.520	5.572
AMDFB	C4	C	X		X	X	X	Jun - 15	Jun - 17	22.089	38.778	28.423	28.501	0.183	0.193	60.787	76.095	48.494	48.592	22.186	4.113	11.905	0.541	9.091	5.520	5.572
AMDFC	C4	C						Jun - 15	Jun - 17	22.089	38.778	28.423	28.501	0.183	0.193	60.787	76.095	48.494	48.592	22.186	4.113	11.905	0.541	9.091	5.520	5.572
JD02A	C5	C	X		X	X	X	Jun - 14	Aug - 17	21.225	39.110	28.566	28.901	0.296	0.254	90.261	73.227	47.767	46.985	3.671	8.370	1.615	3.377	79.736	4.921	5.097
JD02B	C5	C		X	X	X	X	Jun - 14	Aug - 17	21.225	39.110	28.566	28.901	0.296	0.254	90.261	73.227	47.767	46.985	3.671	8.370	1.615	3.377	79.736	4.921	5.097
JD02C	C5	C		X	X	X	X	Jun - 14	Aug - 17	21.225	39.110	28.566	28.901	0.296	0.254	90.261	73.227	47.767	46.985	3.671	8.370	1.615	3.377	79.736	4.921	5.097
JD03A	C6	C						Jun - 14	Apr - 17	21.082	39.200	28.647	29.000	0.301	0.249	83.464	71.550	47.652	46.837	6.285	16.061	3.492	0.140	73.324	5.028	5.675
JD03B	C6	C						Jun - 14	Apr - 17	21.082	39.200	28.647	29.000	0.301	0.249	83.464	71.550	47.652	46.837	6.285	16.061	3.492	0.140	73.324	5.028	5.675
JD03C	C6	C						Jun - 14	Apr - 17	21.082	39.200	28.647	29.000	0.301	0.249	83.464	71.550	47.652	46.837	6.285	16.061	3.492	0.140	73.324	5.028	5.675
ALR5A	C7	C	X	X	X	X	X	May - 15	May - 17	20.496	39.636	28.807	29.150	0.320	0.284	130.623	155.906	48.448	48.223	17.469	47.924	5.501	0.054	24.567	4.655	5.012
ALR5B	C7	C	X	X	X	X	X	May - 15	May - 17	20.496	39.636	28.807	29.150	0.320	0.284	130.623	155.906	48.448	48.223	17.469	47.924	5.501	0.054	24.567	4.655	5.012
ALR5C	C7	C						May - 15	May - 17	20.496	39.636	28.807	29.150	0.320	0.284	130.623	155.906	48.448	48.223	17.469	47.924	5.501	0.054	24.567	4.655	5.012
ALR2A	S1	S						May - 15	May - 17	20.148	40.238	29.413	29.770	0.635	0.614	100.536	106.960	48.064	48.074	57.956	0.105	1.791	0.632	34.352	4.449	4.935
ALR2B	S1	S						May - 15	May - 17	20.148	40.238	29.413	29.770	0.635	0.614	100.536	106.960	48.064	48.074	57.956	0.105	1.791	0.632	34.352	4.449	4.935
ALR2C	S1	S	X	X	X	X	X	May - 15	May - 17	20.148	40.238	29.413	29.770	0.635	0.614	100.536	106.960	48.064	48.074	57.956	0.105	1.791	0.632	34.352	4.449	4.935
ALR7A	S2	S						May - 15	May - 17	20.122	40.218	29.176	29.501	0.391	0.380	119.396	109.856	48.208	48.037	20.277	14.941	16.969	0.213	45.571	4.301	4.832
ALR7B	S2	S	X	X	X	X	X	May - 15	May - 17	20.122	40.218	29.176	29.501	0.391	0.380	119.396	109.856	48.208	48.037	20.277	14.941	16.969	0.213	45.571	4.301	4.832
ALR7C	S2	S						May - 15	May - 17	20.122	40.218	29.176	29.501	0.391	0.380	119.396	109.856	48.208	48.037	20.277	14.941	16.969	0.213	45.571	4.301	4.832
ALR3A	S3	S	X	X	X	X	X	May - 15	May - 17	19.907	40.523	29.663	30.137	0.630	0.632	242.620	281.014	47.901	47.891	47.415	0.220	11.661	1.870	25.963	4.347	4.860
ALR3B	S3	S	X	X	X	X	X	May - 15	May - 17	19.907	40.523	29.663	30.137	0.630	0.632	242.620	281.014	47.901	47.891	47.415	0.220	11.661	1.870	25.963	4.347	4.860
ALR3C	S3	S						May - 15	May - 17	19.907	40.523	29.663	30.137	0.630	0.632	242.620	281.014	47.901	47.891	47.415	0.220	11.661	1.870	25.963	4.347	4.860
R18A	S4	S	X	X	X	X	X	Feb - 15	Nov - 17	16.880	42.263	29.588	29.922	1.745	1.305	699.815	388.480	49.153	49.699	7.302	5.714	16.402	1.058	66.349	5.041	5.157
R18B	S4	S	X	X	X	X	X	Feb - 15	Nov - 17	16.880	42.263	29.588	29.922	1.745	1.305	699.815	388.480	49.153	49.699	7.302	5.714	16.402	1.058	66.349	5.041	5.157
R18C	S4	S	X	X	X	X	X	Feb - 15	Nov - 17	16.880	42.263	29.588	29.922	1.745	1.305	699.815	388.480	49.153	49.699	7.302	5.714	16.402	1.058	66.349	5.041	5.157
R24A	S5	S		X	X	X	X	Feb - 15	Nov - 17	16.791	42.200	29.682	30.046	2.520	2.074	603.443	535.587	48.881	49.420	11.212	0.000	25.481	46.206	4.757	5.053	5.235
R24B	S5	S						Feb - 15	Nov - 17	16.791	42.200	29.682	30.046	2.520	2.074	603.443	535.587	48.881	49.420	11.212	0.000	25.481	46.206	4.757	5.053	5.235
R24C	S5	S	X	X	X	X	X	Feb - 15	Nov - 17	16.791	42.200	29.682	30.046	2.520	2.074	603.443	535.587	48.881	49.420	11.212	0.000	25.481	46.206	4.757	5.053	5.235

Table S2. Pearson correlation between explanatory variables obtained from remote sensing data and benthic surveys. Average over five years before the retrieval date of sea surface temperature (SST), SST differences between hottest and coldest month (SSTvar), chlorophyll a concentrations (Chla), particulate organic carbon concentrations (POC), and photosynthetic active radiation (PAR). Average over the deployment period of sea surface temperature (Dep.SST), SST differences between hottest and coldest month (Dep.SSTvar), chlorophyll a concentrations (Dep.Chla), particulate organic carbon concentrations (Dep.POC), and photosynthetic active radiation (Dep.PAR). Percentage cover of hard corals (HC), soft corals (SC), turf algae (Turf), macroalgae (MA), and not living substrate (Abiotic).

	SST	Dep.SST	Dep.Chla	Chla	POC	Dep.POC	PAR	Dep.PAR	HC	SC	Turf	MA	Abiotic	SSTvar	Dep.SSTvar
SST															
Dep.SST	1														
Dep.Chla	0.59	0.58													
Chla	0.59	0.58	1												
POC	0.52	0.52	0.89	0.92											
Dep.POC	0.57	0.58	0.93	0.93	0.94										
PAR	0.85	0.82	0.59	0.61	0.54	0.52									
Dep.PAR	0.73	0.7	0.72	0.73	0.68	0.66	0.93								
HC	0.05	0.03	0.06	0.12	0.19	0.08	0.17	0.09							
SC	0.04	0.06	0.31	0.27	0.2	0.22	0.11	0.06	0.24						
Turf	0.23	0.27	0.12	0.11	0.09	0.11	0.02	0.18	0.28	0.32					
MA	0.34	0.35	0.82	0.8	0.59	0.75	0.35	0.43	0.18	0.2	0.12				
Abiotic	0.09	0.07	0.28	0.23	0.07	0.29	0.22	0.38	0.47	0.04	0.21	0.4			
SSTvar	-0.92	-0.93	-0.32	-0.32	-0.27	-0.32	-0.75	-0.54	-0.04	-0.24	0.44	-0.18	-0.01		
Dep.SSTvar	-0.94	-0.94	-0.38	-0.37	-0.31	-0.36	-0.82	-0.62	-0.01	-0.20	0.38	-0.21	0.03	0.99	

Table S3. Significance values of the differences of satellite derived products and percentage cover of ecologically meaningful benthic structures between regions. A subsample of 15 ARMS per region was taken to homogenize the sampling size. Data was not normally distributed in each region; therefore, the non-parametric Kruskal-Wallis test was used.

	Unit	North mean	Central mean	South mean	Chi-Square	P Kruskal Wallis
SST	C	26.95	28.60	29.50	0.32	p<0.01
PAR	Einstein m-2 d-1	46.89	48.29	48.44	18.08	p<0.01
Chla	mg m $^{-3}$	0.27	0.37	1.18	14.13	p<0.01
HC	%cover	23.90	13.19	28.83	3.87	p=0.14
MA	%cover	0.33	0.88	10.00	7.98	p=0.02
SC	%cover	5.85	13.71	4.20	4.22	p=0.12
Turf	%cover	20.00	20.52	14.46	1.06	p=0.59
Abiotic	%cover	43.28	38.52	35.40	0.32	p=0.85

Table S4. The 10 highest averages of the OTUs influencing differences in community structure obtained using a Simper analysis with 999 permutations and their respective taxonomy.

North and Central										
Rank	OTU Number	Taxonomy	average	sd	ratio	ava	avb	cumsum	p	
1	OTU_123	*Exoclimenella*	0.0815501	0.080561	1.0123	5.38889	3.66667	0.09379	0.006	**
2	OTU_69	Galatheidae	0.0639615	0.086783	0.737	6.27778	0.19048	0.16736	0.001	***
3	OTU_47	*Palaemonella pottsi*	0.0370032	0.036204	1.0221	2.94444	1.61905	0.20992	0.981	
4	OTU_103	*Thalamitoides*	0.0308066	0.029578	1.0415	2.94444	2.57143	0.24535	0.727	
5	OTU_73	Paguroidea	0.0271638	0.039497	0.6878	2.88889	0	0.27659	0.193	
6	OTU_48	Gastropoda	0.0264053	0.031582	0.8361	2	1.09524	0.30696	0.01	**
7	OTU_1	Paguroidea	0.0259286	0.025707	1.0086	2.22222	0.95238	0.33678	0.459	
8	OTU_83	Paguroidea	0.025486	0.045751	0.5571	1.16667	2.52381	0.36609	0.637	
9	OTU_60	Decapoda	0.0253889	0.036655	0.6927	1.5	1.71429	0.3953	0.018	*
10	OTU_50	Paguroidea	0.0200064	0.021601	0.9262	1.5	0.61905	0.41831	0.001	***
North and South										
Rank	OTU Number	Taxonomy	average	sd	ratio	ava	avb	cumsum	p	
1	OTU_123	*Exoclimenella*	0.0542662	0.050334	1.0781	5.38889	3.6	0.06647	0.924	
2	OTU_47	*Palaemonella pottsi*	0.0524508	0.049658	1.0562	2.94444	6.06667	0.13072	0.275	
3	OTU_69	Galatheidae	0.0518292	0.070263	0.7376	6.27778	0.46667	0.1942	0.054	.
4	OTU_3	*Ophiothrix*	0.0332603	0.05655	0.5882	1.72222	4.26667	0.23494	0.062	.
5	OTU_73	Paguroidea	0.0310835	0.035926	0.8652	2.88889	1.8	0.27301	0.072	.
6	OTU_103	*Thalamitoides*	0.0262518	0.02341	1.1214	2.94444	1.93333	0.30517	0.958	
7	OTU_1	Paguroidea	0.0247483	0.020732	1.1937	2.22222	2.26667	0.33548	0.664	
8	OTU_78	*Phylladiorhynchus*	0.0198213	0.025146	0.7882	0.77778	1.93333	0.35976	0.707	
9	OTU_63	*Luniella spinipes*	0.0192952	0.016379	1.1781	0.33333	2.2	0.3834	0.002	**
10	OTU_83	Paguroidea	0.0173807	0.022362	0.7772	1.16667	1.46667	0.40469	0.896	
Central and South										
Rank	OTU Number	Taxonomy	average	sd	ratio	ava	avb	cumsum	p	
1	OTU_47	*Palaemonella pottsi*	0.0704751	0.084339	0.8356	1.61905	6.06667	0.08013	0.002	**
2	OTU_123	*Exoclimenella*	0.0697448	0.067986	1.0259	3.66667	3.6	0.15943	0.247	
3	OTU_1	Paguroidea	0.0344942	0.036168	0.9537	0.95238	2.26667	0.19865	0.003	**
4	OTU_78	*Phylladiorhynchus*	0.0329544	0.048391	0.681	1.19048	1.93333	0.23612	0.002	**
5	OTU_103	*Thalamitoides*	0.0326047	0.032915	0.9906	2.57143	1.93333	0.27319	0.504	
6	OTU_83	Paguroidea	0.0320068	0.046868	0.6829	2.52381	1.46667	0.30958	0.183	
7	OTU_3	*Ophiothrix*	0.0311136	0.069527	0.4475	0.33333	4.26667	0.34496	0.15	
8	OTU_63	*Luniella spinipes*	0.0272565	0.024805	1.0988	0.09524	2.2	0.37595	0.001	***
9	OTU_73	Paguroidea	0.0223604	0.043699	0.5117	0	1.8	0.40137	0.653	
10	OTU_126	*Galathea*	0.0182542	0.02425	0.7527	1.14286	0.13333	0.42213	0.083	.

Figure S1.- Venn diagram showing the number of operational taxonomic units (OTUs) unique to the central region and Al Lith (S1, S2, and S3) and Farasan Islands reefs (S4 and S5) and shared between them using a random subsample of 9 ARMS of the central region.

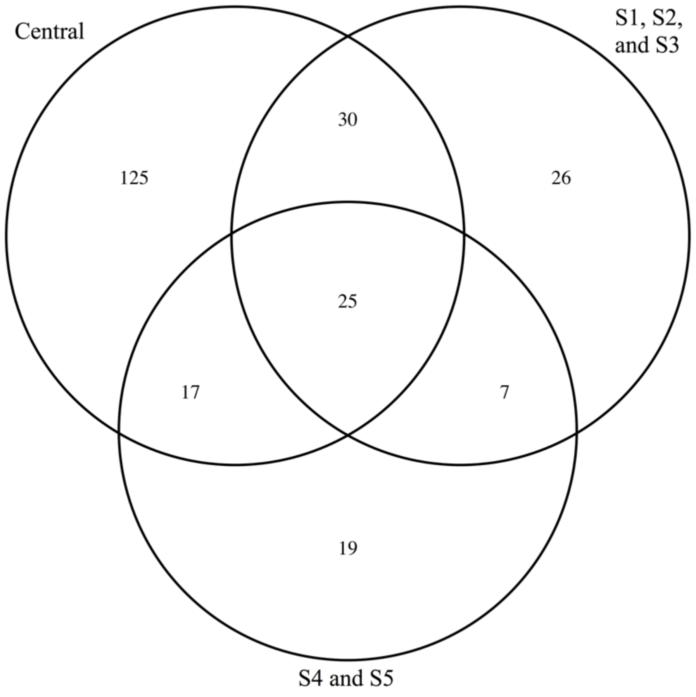

Figures S2 to S12.- CART models using regression tree to distinguish critical breakpoints in the abundance of the OTUs that significantly contributed to the dissimilarities observed. Sea surface temperature values (Av5yr.SST in Celsius), chlorophyll – a (Av5yr.Chla in mg m^{-3}), particulated organic carbon (Av5yr.POC in mg m^{-3}), and photosynthetically active radiation (Av5yr.PAR in Einstein m^{-2} d^{-1}) are averages of the 5 years before the retrieval of the ARMS. Percentage cover of Hard coral (HC), soft coral (SC), turf algae (Turf), macro algae (MA), and non-live substrate (Abiotic) were obtained from benthic surveys.

S2

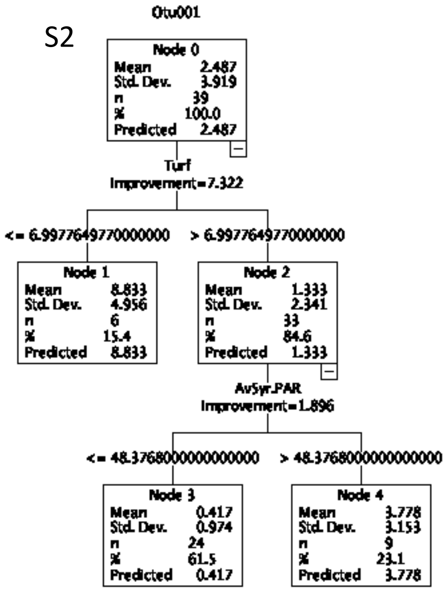

S3

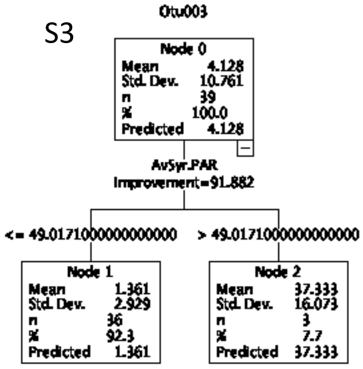

S4

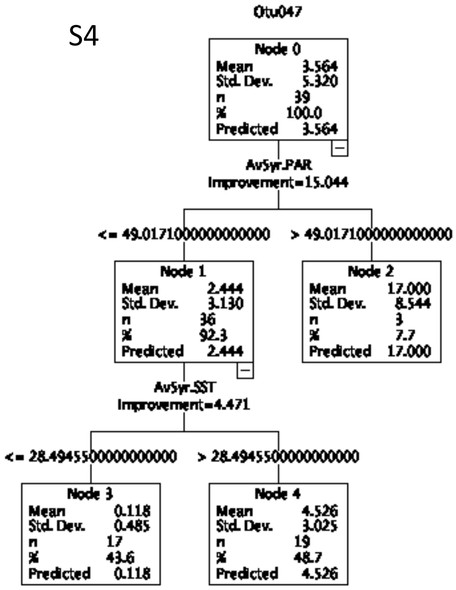
Otu047
Node 0
Mean 3.564
Std. Dev. 5.320
n 39
% 100.0
Predicted 3.564
AvSyr.PAR
Improvement=15.044
<= 49.0171000000000000
> 49.0171000000000000
Node 1
Mean 2.444
Std. Dev. 3.130
n 36
% 92.3
Predicted 2.444
Node 2
Mean 17.000
Std. Dev. 8.544
n 3
% 7.7
Predicted 17.000
AvSyr.SST
Improvement=4.471
<= 28.4945500000000000
> 28.4945500000000000
Node 3
Mean 0.118
Std. Dev. 0.485
n 17
% 43.6
Predicted 0.118
Node 4
Mean 4.526
Std. Dev. 3.025
n 19
% 48.7
Predicted 4.526

S5

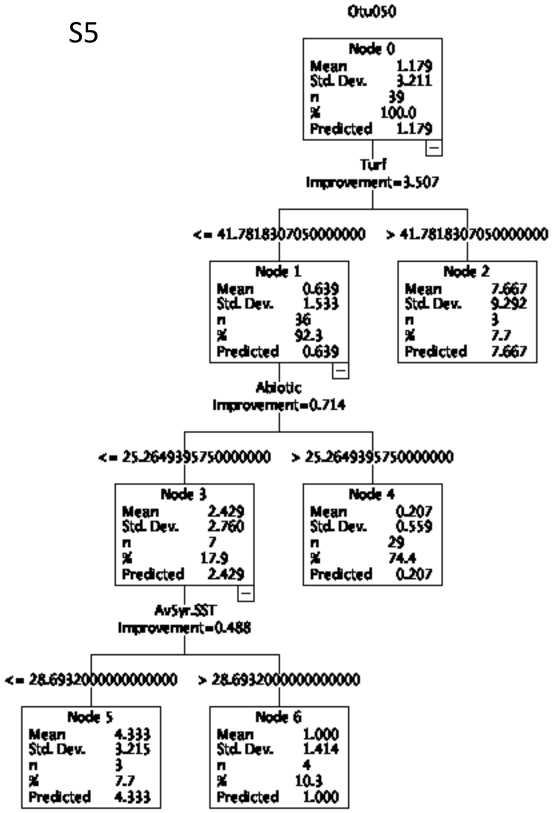
Otu050
Node 0
Mean 1.179
Std. Dev. 3.211
n 39
% 100.0
Predicted 1.179
Turf
Improvement=3.507
<= 41.7818307050000000
> 41.7818307050000000
Node 1
Mean 0.639
Std. Dev. 1.533
n 36
% 92.3
Predicted 0.639
Node 2
Mean 7.667
Std. Dev. 9.292
n 3
% 7.7
Predicted 7.667
Abiotic
Improvement=0.714
<= 25.2649395750000000
> 25.2649395750000000
Node 3
Mean 2.429
Std. Dev. 2.760
n 7
% 17.9
Predicted 2.429
Node 4
Mean 0.207
Std. Dev. 0.559
n 29
% 74.4
Predicted 0.207
AvSyr.SST
Improvement=0.488
<= 28.6932000000000000
> 28.6932000000000000
Node 5
Mean 4.333
Std. Dev. 3.215
n 3
% 7.7
Predicted 4.333
Node 6
Mean 1.000
Std. Dev. 1.414
n 4
% 10.3
Predicted 1.000

S6

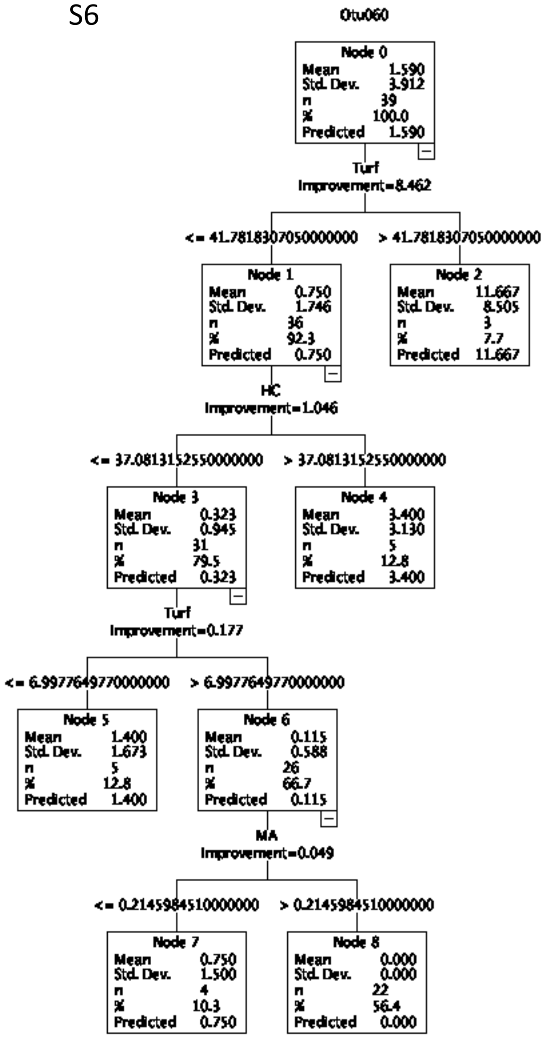
Otu060
Node 0
Mean 1.590
Std. Dev. 3.912
n 39
% 100.0
Predicted 1.590
Turf
Improvement=8.462
<= 41.7818307050000000
> 41.7818307050000000
Node 1
Mean 0.750
Std. Dev. 1.746
n 36
% 92.3
Predicted 0.750
Node 2
Mean 11.667
Std. Dev. 8.505
n 3
% 7.7
Predicted 11.667
HC
Improvement=1.046
<= 37.0813152550000000
> 37.0813152550000000
Node 3
Mean 0.323
Std. Dev. 0.945
n 31
% 79.5
Predicted 0.323
Node 4
Mean 3.400
Std. Dev. 3.130
n 5
% 12.8
Predicted 3.400
Turf
Improvement=0.177
<= 6.9977649770000000
> 6.9977649770000000
Node 5
Mean 1.400
Std. Dev. 1.673
n 5
% 12.8
Predicted 1.400
Node 6
Mean 0.115
Std. Dev. 0.588
n 26
% 66.7
Predicted 0.115
MA
Improvement=0.049
<= 0.2145984510000000
> 0.2145984510000000
Node 7
Mean 0.750
Std. Dev. 1.500
n 4
% 10.3
Predicted 0.750
Node 8
Mean 0.000
Std. Dev. 0.000
n 22
% 56.4
Predicted 0.000

S7

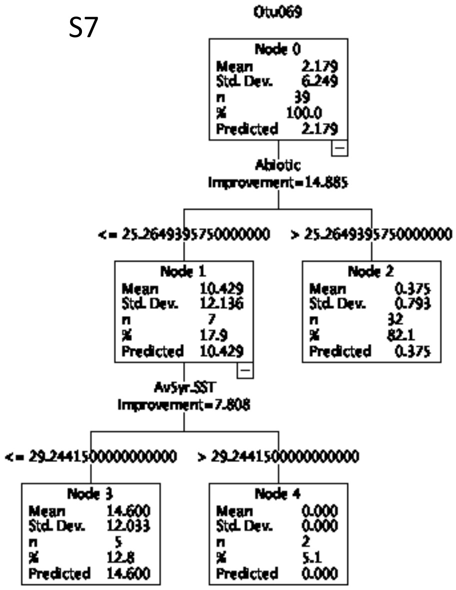
Otu069
Node 0
Mean 2.179
Std. Dev. 6.249
n 39
% 100.0
Predicted 2.179
Abiotic
Improvement=14.885
<= 25.2649395750000000
> 25.2649395750000000
Node 1
Mean 10.429
Std. Dev. 12.136
n 7
% 17.9
Predicted 10.429
Node 2
Mean 0.375
Std. Dev. 0.793
n 32
% 82.1
Predicted 0.375
AvSyr.SST
Improvement=7.808
<= 29.2441500000000000
> 29.2441500000000000
Node 3
Mean 14.600
Std. Dev. 12.033
n 5
% 12.8
Predicted 14.600
Node 4
Mean 0.000
Std. Dev. 0.000
n 2
% 5.1
Predicted 0.000

S8

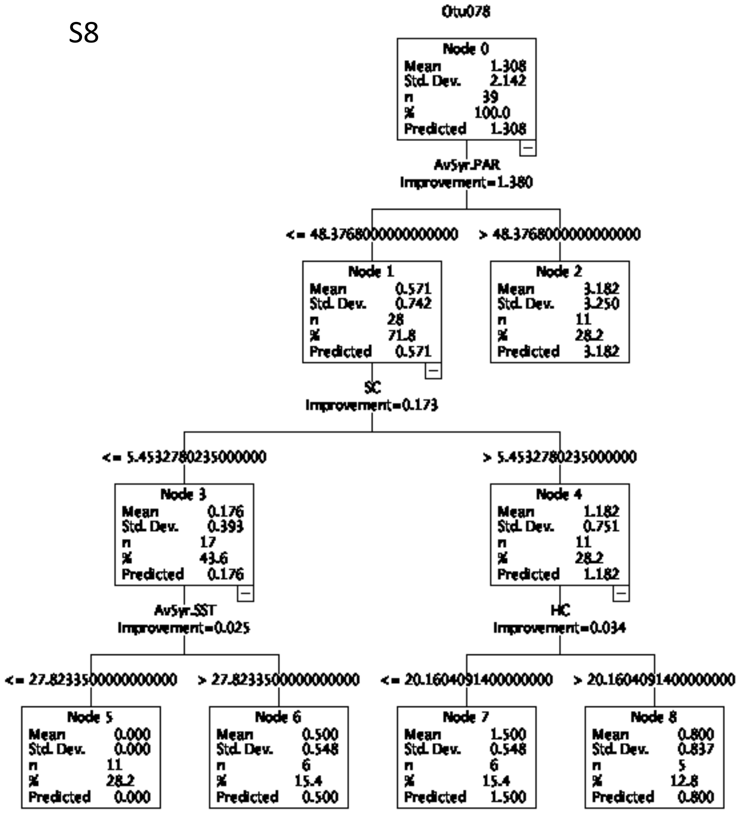
Otu078
Node 0
Mean 1.308
Std. Dev. 2.142
n 39
% 100.0
Predicted 1.308
AvSyr.PAR
Improvement=1.380
<= 48.3768000000000000
> 48.3768000000000000
Node 1
Mean 0.571
Std. Dev. 0.742
n 28
% 71.8
Predicted 0.571
Node 2
Mean 3.182
Std. Dev. 3.250
n 11
% 28.2
Predicted 3.182
SC
Improvement=0.173
<= 5.4532780235000000
> 5.4532780235000000
Node 3
Mean 0.176
Std. Dev. 0.393
n 17
% 43.6
Predicted 0.176
Node 4
Mean 1.182
Std. Dev. 0.751
n 11
% 28.2
Predicted 1.182
AvSyr.SST
Improvement=0.025
<= 27.8233500000000000
> 27.8233500000000000
HC
Improvement=0.034
<= 20.1604091400000000
> 20.1604091400000000
Node 5
Mean 0.000
Std. Dev. 0.000
n 11
% 28.2
Predicted 0.000
Node 6
Mean 0.500
Std. Dev. 0.548
n 6
% 15.4
Predicted 0.500
Node 7
Mean 1.500
Std. Dev. 0.548
n 6
% 15.4
Predicted 1.500
Node 8
Mean 0.800
Std. Dev. 0.837
n 5
% 12.8
Predicted 0.800

S9

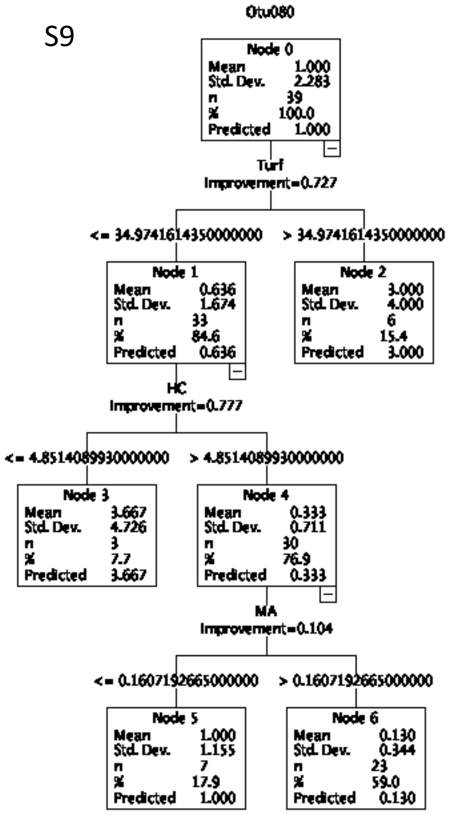
Otu080
Node 0
Mean 1.000
Std. Dev. 2.283
n 39
% 100.0
Predicted 1.000
Turf
Improvement=0.727
<= 34.9741614350000000
> 34.9741614350000000
Node 1
Mean 0.636
Std. Dev. 1.674
n 33
% 84.6
Predicted 0.636
Node 2
Mean 3.000
Std. Dev. 4.000
n 6
% 15.4
Predicted 3.000
HC
Improvement=0.777
<= 4.8514089930000000
> 4.8514089930000000
Node 3
Mean 3.667
Std. Dev. 4.726
n 3
% 7.7
Predicted 3.667
Node 4
Mean 0.333
Std. Dev. 0.711
n 30
% 76.9
Predicted 0.333
MA
Improvement=0.104
<= 0.1607192665000000
> 0.1607192665000000
Node 5
Mean 1.000
Std. Dev. 1.155
n 7
% 17.9
Predicted 1.000
Node 6
Mean 0.130
Std. Dev. 0.344
n 23
% 59.0
Predicted 0.130

S10

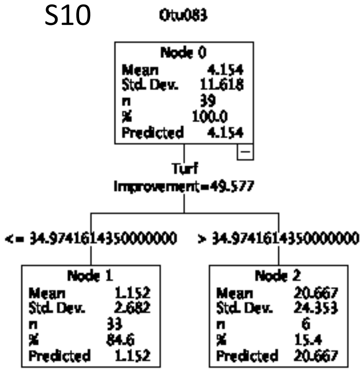
Otu083
Node 0
Mean 4.154
Std. Dev. 11.618
n 39
% 100.0
Predicted 4.154
Turf
Improvement=49.577
<= 34.9741614350000000
> 34.9741614350000000
Node 1
Mean 1.152
Std. Dev. 2.682
n 33
% 84.6
Predicted 1.152
Node 2
Mean 20.667
Std. Dev. 24.353
n 6
% 15.4
Predicted 20.667

S11

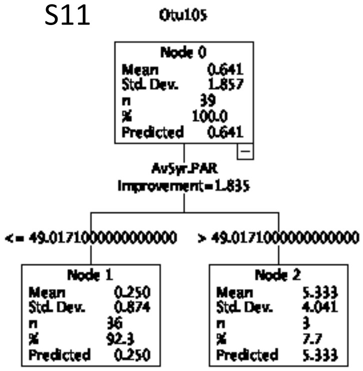
Otu105
Node 0
Mean 0.641
Std. Dev. 1.857
n 39
% 100.0
Predicted 0.641
AvSyr.PAR
Improvement=1.835
<= 49.0171000000000000
> 49.0171000000000000
Node 1
Mean 0.250
Std. Dev. 0.874
n 36
% 92.3
Predicted 0.250
Node 2
Mean 5.333
Std. Dev. 4.041
n 3
% 7.7
Predicted 5.333

S12

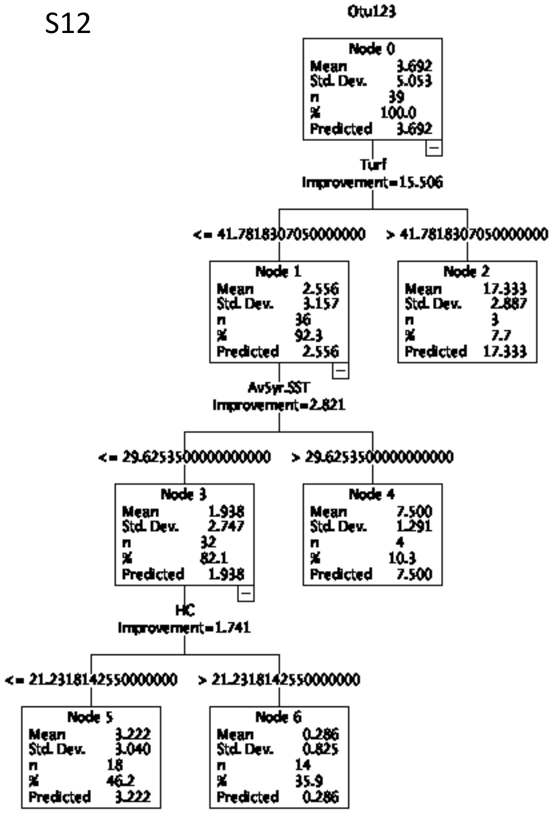
Otu123
Node 0
Mean 3.692
Std. Dev. 5.053
n 39
% 100.0
Predicted 3.692
Turf
Improvement=15.506
<= 41.7818307050000000
> 41.7818307050000000
Node 1
Mean 2.556
Std. Dev. 3.157
n 36
% 92.3
Predicted 2.556
Node 2
Mean 17.333
Std. Dev. 2.887
n 3
% 7.7
Predicted 17.333
AvSyr.SST
Improvement=2.821
<= 29.6253500000000000
> 29.6253500000000000
Node 3
Mean 1.938
Std. Dev. 2.747
n 32
% 82.1
Predicted 1.938
Node 4
Mean 7.500
Std. Dev. 1.291
n 4
% 10.3
Predicted 7.500
HC
Improvement=1.741
<= 21.2318142550000000
> 21.2318142550000000
Node 5
Mean 3.222
Std. Dev. 3.040
n 18
% 46.2
Predicted 3.222
Node 6
Mean 0.286
Std. Dev. 0.825
n 14
% 35.9
Predicted 0.286
